# Investigations of Antioxidant and Anti-Cancer Activities of 5-Aminopyrazole Derivatives

**DOI:** 10.3390/molecules29102298

**Published:** 2024-05-14

**Authors:** Federica Rapetti, Andrea Spallarossa, Eleonora Russo, Debora Caviglia, Carla Villa, Bruno Tasso, Maria Grazia Signorello, Camillo Rosano, Erika Iervasi, Marco Ponassi, Chiara Brullo

**Affiliations:** 1 Department of Pharmacy (DIFAR), University of Genoa, Viale Benedetto XV, 3, 16132 Genoa, Italy; federica.rapetti@unige.it (F.R.); andrea.spallarossa@unige.it (A.S.); eleonora.russo@unige.it (E.R.); debora.caviglia@edu.unige.it (D.C.); carla.villa@unige.it (C.V.); bruno.tasso@unige.it (B.T.); 2 Department of Pharmacy (DIFAR), Biochemistry Lab., University of Genoa, Viale Benedetto XV, 3, 16132 Genova, Italy; mariagrazia.signorello@unige.it; 3 IRCCS Ospedale Policlinico San Martino, Proteomics and Mass Spectrometry Unit, L.go. R. Benzi, 10, 16132 Genova, Italy; camillo.rosano@hsanmartino.it (C.R.); iervasierika@gmail.com (E.I.); marco.ponassi@hsanmartino.it (M.P.)

**Keywords:** 5-aminopyrazoles, MTT test, anti-cancer activity, DPPH, ROS, druglike properties

## Abstract

To further extend the structure-activity relationships (SARs) of 5-aminopyrazoles (5APs) and identify novel compounds able to interfere with inflammation, oxidative stress, and tumorigenesis, 5APs **1–4** have been designed and prepared. Some chemical modifications have been inserted on cathecol function or in aminopyrazole central core; in detail: (i) smaller, bigger, and more lipophilic substituents were introduced in *meta* and *para* positions of catechol portion (5APs **1**); (ii) a methyl group was inserted on C3 of the pyrazole scaffold (5APs **2**); (iii) a more flexible alkyl chain was inserted on N1 position (5APs **3**); (iv) the acylhydrazonic linker was moved from position 4 to position 3 of the pyrazole scaffold (5APs **4**). All new derivatives **1–4** have been tested for radical scavenging (DPPH assay), anti-aggregating/antioxidant (in human platelets) and cell growth inhibitory activity (MTT assay) properties. In addition, in silico pharmacokinetics, drug-likeness properties, and toxicity have been calculated. 5APs **1** emerged to be promising anti-proliferative agents, able to suppress the growth of specific cancer cell lines. Furthermore, derivatives **3** remarkably inhibited ROS production in platelets and 5APs **4** showed interesting in vitro radical scavenging properties. Overall, the collected results further confirm the pharmaceutical potentials of this class of compounds and support future studies for the development of novel anti-proliferative and antioxidant agents.

## 1. Introduction

Pyrazole nucleus has been extensively investigated as pharmacophore [1,2,3,4,5,6,7,8,9,10,11] and several aminopyrazoles bearing a free amino group showed relevant biological activity in different therapeutic areas [12,13,14,15,16]. Specifically, 5-aminopyrazoles (5APs) have been deeply studied for their anti-inflammatory and anticancer activity [17,18], providing useful ligands for receptors or enzymes, as p38 MAPK [19,20,21,22], COX [23], carbonic anhydrase [24], and other different targets involved in cancer progression [25,26], as demonstrated by the recent approval of 5AP **Pirtobrutinib**, a reversible BTK inhibitor, clinically used for the treatment of mantle cell lymphoma (MCL) [27,28,29,30,31,32,33].

**Figure 1:**
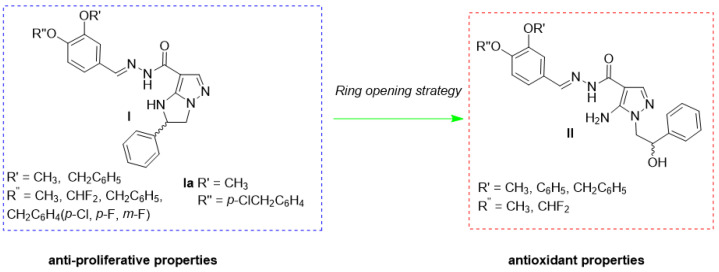
Chemical structure of previous compounds **I** and derived 5APs **II**.

In the effort to synthetize new pyrazole-based compounds able to interfere with inflammation, oxidative stress, and tumorigenesis, we recently reported derivatives **I** (Figure 1) endowed with excellent anti-proliferative activity. Molecular docking and molecular dynamic simulations suggested the ability of the most active compound **Ia** (Figure 1) to interact with polymeric tubulin α/tubulin β/stathmin4 complex at the colchicine binding site [34]. By applying a ring opening strategy, we obtained 5APs **II** (Figure 1), able to strongly inhibit ROS and superoxide anion production, lipid peroxidation, and NADPH oxidase in thrombin-stimulated human platelets and ROS formation in EAhy926 cells [35]. 

**Figure 2:**
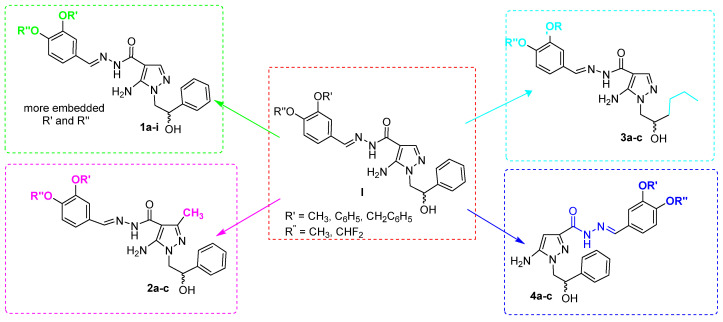
Chemical structure of previous 5-APs **I** and new 5APs **1–4**.

To further extend the structure-activity relationships (SARs) of this class of compounds and verify their biological profile, a new library (eighteen compounds) of 5APs **1–4** was designed and synthesized (Figure 2 and Table 1). In detail: (i) in 5APs **1,** smaller, bigger, and more lipophilic substituents, similar to previous **II**, were introduced in *meta* and *para* positions of catechol portion; (ii) in 5APs **2**, a methyl group on C3 of the pyrazole scaffold was inserted to increase steric hindrance; (iii) in 5APs **3**, a more flexible alkyl chain was inserted on N1 position; (iv) finally, in 5APs **4**, the acylhydrazonic substituent was shifted from position 4 to position 3 of the pyrazole scaffold.

With the aim to investigate the antioxidant and anti-cancer activity of this novel library, in analogy with previous derivatives **I** and **II**, all new derivatives **1–4** have been tested for: (i) in vitro radical-scavenging activity (DPPH test); (ii) anti-aggregating/antioxidant activity in human platelets; and (iii) cell growth inhibitory activity. In addition, in silico pharmacokinetics, drug-likeness properties, and toxicity have been calculated. 

## 2. Results

### 2.1. Chemistry

**Scheme 1:**
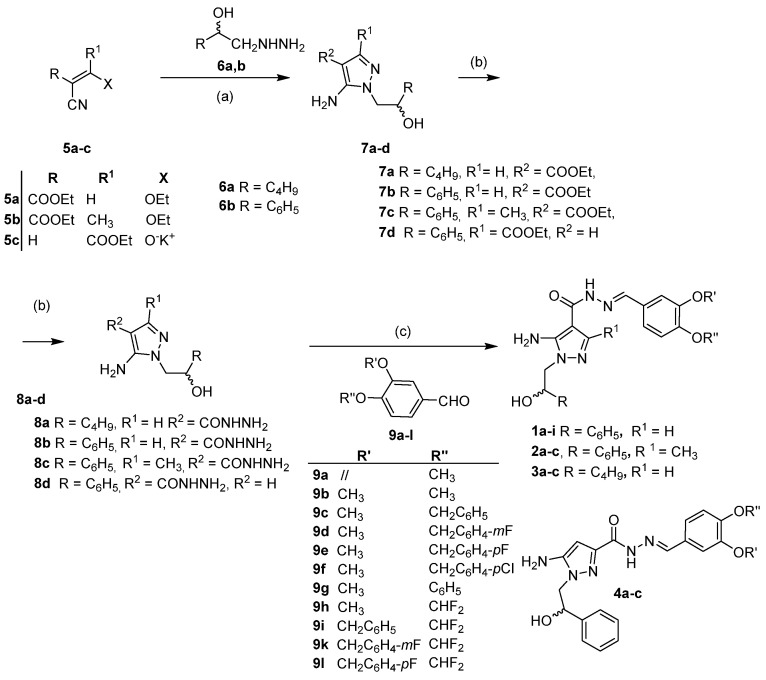
Synthesis of compounds **1–4**. Reagents and conditions: (a) Toluene, 70–80 °C, 8 h (**7b**, **7d**) or absolute ethanol (abs. EtOH), reflux (**7a**, **7c**); (b) NH_2_NH_2_·H_2_O, 120–130 °C, 3–4 h (**8a–c**) or r.t., 6 h (**8d**); (c) abs. EtOH, **9a–l**, reflux 1–18 h.

5APs **1–4** were synthesized in good-to-moderate yields through the three-step sequential procedure (Scheme 1). Briefly, the condensation of the proper ethyl cyanoacrylate **5a–c** with suitable hydrazinoethanol **6a,b** [36,37,38] led to 5APs **7a–d**, as previously reported [37,38,39]. These intermediates were then transformed in the corresponding hydrazides **8a–d** by reaction with hydrazine monohydrate. Finally, the reaction between carbohydrazide intermediates **8** and the suitable benzaldehydes **9a–l** (commercially available or prepared via literature methods [40,41]) allowed the isolation of the desired compounds **1–4** (Table 1). 

As previously reported, the final reaction proved to be stereoselective and only hydrazones *E* were isolated, as assessed by NMR spectral analyses [34].

### 2.2. In Vitro Antioxidant Activity (DPPH Assay)

The antioxidant activity of the new synthesized compounds was measured in vitro using the 2,2-diphenyl-1-picrylhydrazyl (DPPH) assay [42,43]. The results were calculated as Trolox equivalent and expressed as percentage of antioxidant activity (AA%) (Table 2). The best AA% values have been obtained for **4b** and **4c** (27.65 and 15.47%, respectively); intermediate activity was shown by **1d**, **1h**, **1i**, **2b**, **2c**, **3c**, and **4a** (AA% range = 4.22–6.12%), while all other tested compounds resulted ineffective.

### 2.3. Inhibiting Effect on Human Platelet Aggregation and ROS Production

As previously reported for compounds **I** and **II**, platelets could be considered inflammatory cells and could represent a simple, economic, and suitable cellular model based on a causal relationship between inflammation and tumorigenesis [34]. Inflammation and thrombosis are two critical, closely interconnected processes in the response to injury and infection. This correlation has long been recognized, particularly in atherosclerotic cardiovascular disease [44] as well in cancer [45]. 

Oxidative stress has been associated with several pathological conditions (e.g., cancer, diabetes, metabolic disorders, atherosclerosis, and cardiovascular diseases) [46] and plays a significant role in promoting inflammation and platelet activation [47]. Thus, we tested the new 5AP library on human platelets to verify the inhibitory activity on aggregation and ROS production. As reported in Table 3, the newly synthetized compounds affected platelet aggregation and ROS production. Thus, the majority of the tested derivatives displayed IC_50_ ≤ 200 μM on both tested parameters; **3b** and **3c** showed the higher inhibitory properties against both platelet aggregation and ROS production (IC_50_ values between 113 and 139 μM). It should also be noted that three compounds have intermediate IC_50_ values (around 400 μM), while five are virtually inactive. 

### 2.4. Cell Growth Inhibitory Activity

APs **1–4** were tested for anti-proliferative activity on a panel of 60 different cancer cell lines by National Cancer Institute (Developmental Therapeutics Program, Division of Cancer Treatment and Diagnosis, Table 4) [48]. 

Compounds **2** (3-methyl substituted pyrazoles) and **4** (3-benzylidenecarbohydrazide pyrazoles) did not show a relevant anti-cancer effect, thus indicating that 3-unsubstitued pyrazole scaffold is as key determinant for anti-proliferative activity. Conversely, compounds **1**, close analogues of previous **II**, and derivatives **3**, characterized by a more flexible chain on N1, evidenced a good percentage of cancer cell growth inhibition in different cancer cell lines (Table 4). 

Interestingly, among compounds **1**, only derivatives with hindered substituents on catechol portion (i.e., OPh, OCH_2_Ar) and more strictly related to previous **I** showed growth percent inhibition values lower than 40% against selected cancer cell lines (**1c** and **1f** on breast cancer cell lines; **1d** on leukemia cell lines and breast cancer cell lines, **1e** on renal cell lines; Table 4). Remarkably, compound **1e** proved to selectively block the growth of renal cancer cell line CAKI-1.

Within series **3**, compounds **3a** and **3c** showed a relevant anti-proliferative activity against different leukemic cell lines. 

The significant cell growth inhibitory activity of **1c**, **1d**, **1f,** and **1g** against breast cancer cell lines prompted us to further evaluate (at fixed concentration of 10 µM, MTT assay) their effect against other breast adenocarcinoma cancer cell lines (namely, MCF7, MDA-MB231, and SK-BR3) using Cisplatin as reference compound (Table 5). 5-APs **1c** and **1d** were inactive against all three cell lines, **1f** selectively inhibited SK-BR3 cell lines (65% of cell growth), while **1g** showed remarkable action against all breast cancer cell lines, particularly against SKBR3, evidencing a GI_50_ value lower than reference compound Cisplatin (14.4 μM versus 26 μM).

### 2.5. Pharmacokinetic Properties, Druglikness and Toxicity Prediction

To evaluate the pharmaceutical relevance of this new library of 5APs, the pharmacokinetics and drug-likeness properties of all compounds were calculated by SwissADME (Tables S1 and S2, Supporting Information) [49]. 

Collectively the considered compounds are characterized by eight to twelve rotatable bonds, five to eight H-bond acceptors, three H-bond donors, and TPSA values of 123.99 A^2^ except **1a** and **2a** (114.76 A^2^), thus supporting a good capacity of all derivatives to permeate lipophilic barriers. In detail, none of the novel 5APs are able to pass brain-blood barrier (BBB), whereas gastrointestinal (GI) absorption is predicted high for derivatives **1a–g**, **2a**,**3a**, and **4a**. Except for **1i**, all 5APs are predicted to be moderately soluble with a further gain in water solubility for derivatives **3a,b** (ESOL method) [50]. A Lipinski violation (MW > 500 Da) was identified for compounds **1e–i**, **2b**, **2c**, **3c**, **4b**, and **4c**, but no compound showed any pan-assay interference compound (PAINS) alerts. Finally, the presence of a C=N functionality was identified as a limitation, according to the Brenk filter [51].

In addition, the toxicity profiles of all novel compounds were predicted using ProTox webserver (Table S3, Supporting Information) [52,53]. According to the simulation, all APs were predicted to belong to toxicity class 5 (predicted LD_50_ of 4540 mg/kg), with the exception of compounds **1g**, **1i**, and **2a** (predicted LD_50_ = 1000 mg/kg, class 4) and derivative **4c** (LD_50_ = 6000 mg/kg, class 6). No novel derivatives would show any hepatotoxicity, cardiotoxicity, and nephrotoxicity [54], but for most of them (except **1a**, **2c**, and **3a–c**), immunotoxicity (B cell growth inhibition) has been predicted [55]. Finally, no toxicity targets pharmacophores (Novartis off-targets, Adenosine A2a receptor, Adrenergic beta 2 receptor, Androgen receptor, Amine oxidase A, Corticotropin-releasing hormone receptor 1, Dopamine D3 receptor, Estrogen receptor 1, Estrogen receptor 2, Glucocorticoid receptor, Histamine H1 receptor, Nuclear receptor subfamily 1 group I member 2, Opiod receptor kappa 1, Progesterone receptor, Phosphodiesterase 4D, Prostaglandin G/H synthase 1) have been detected. 

## 3. Discussion and Conclusions

**Figure 3:**
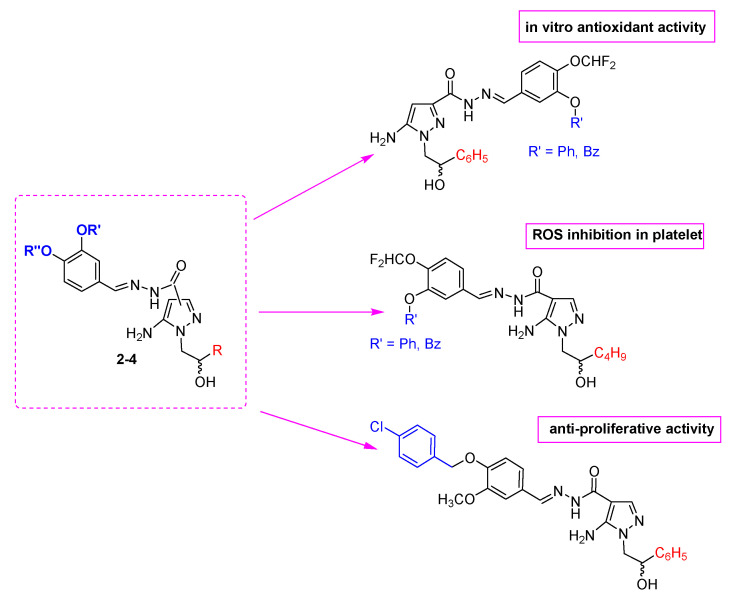
Schematic representation of SARs of novel 5APs here reported.

The biological results reported here pointed at 5AP scaffold as an interesting chemotype to obtain new potential antioxidant/anti-inflammatory/anti-proliferative agents. In detail, the following SARs have been defined (Figure 3):

(i) The presence of the acylhydrazone moiety at position 3 of the pyrazole core increases the radical scavenging activity (compare **4** with **1–3**);
(ii) The best ROS inhibitors in human platelets are 5APs **3** (particularly **3b** and **3c**, with IC_50_ values of 113–115 μM), characterized in N1 by a more flexible alkyl chain (hydroxyhexyl). Furthermore, the substituents on the cathecol portions of these compounds (difluoromethoxy in *para* position and phenoxy or benzyloxy in *meta* position) are the same of **II**, previously identified as potent ROS inhibitors. In addition, **3c** showed significative anti-cancer profile against different leukemic cell lines. These data confirmed the key role of a flexible hydroxyalkyl chain on N1 position, not only for ROS production inhibition, but also for anti-cancer activity;
(iii) 5APs **1** showed promising anti-proliferative activity, probably related to the chemical similarity of these compounds with previous derivatives **I**. Of note is the fact that **1g** (active against breast cancer cell lines) bears the same substituents on the catechol fragment of its precursor **Ia**.
(iv) Finally, compounds **2**, bearing an additional methyl group on C3 position of pyrazole nucleus, did not show a relevant biological activity, confirming that the increase of steric hindrance in this position is detrimental not only for antioxidant activity and ROS production inhibition in platelets but also for anti-proliferative activity.

Collectively, the results here reported extend the SARs on 5AP scaffold, confirming its role as important chemo-type to obtain compounds able to counteract cancer and inflammation.

## 4. Materials and Methods

### 4.1. Chemical Part 

#### 4.1.1. General Information

Chiminord (Milan, Italy) and Aldrich Chemical (Milan, Italy) purchased all chemicals. Solvents were reagent grade. Unless otherwise stated, all commercial reagents were used without further purification. Organic solutions were dried over anhydrous sodium sulphate. A thin layer chromatography (TLC) system was used for routine monitoring of the course of reactions and confirming the purity of analytical samples. Detection of spots was performed using UV light. Merck silica gel, 230–400 mesh, was used for chromatography. Flash chromatography was performed using Isolera one instrument (Biotage, Uppsala, Sweden) using Silicagel column. Melting points are not “corrected” and were measured with a Buchi M-560 instrument (Buchi instruments, Flawil, Switzerland). NMR spectra were recorded on JEOL JNM-ECZR (400 MHz, Tokyo, Japan) instruments (Figures S1–S42, Supporting Information) using CDCl_3_ or DMSO-d_6_ as solvent; chemical shifts are reported as δ (ppm) and signals were characterized as s (singlet), d (doublet), t (triplet), n t (near triplet), q (quartet), m (multiplet), br s (broad signal); J are reported in Hz. 

Elemental analysis was determined with an elemental analyzer EA 1110 (Fison-Instruments, Milan, Italy) and the purity of all synthesized compounds was >95%; products are considered pure when the difference between calculated and found values is ± than 0.4.

#### 4.1.2. Synthesis

Synthesis of compounds **7a–d** and **8b** are yet reported [34,37,38].

##### Synthesis of Carbohydrazide **8a**, **8c**, and **8d**

Suitable compound **7** (5 mmol) and hydrazine monohydrate (0.25 g, 0.25 mL, 5 mmol) are heated to 120–130 °C for 4 h (**8a** and **8c**) or at room temperature for 6 h (**8d**). After cooling to room temperature, H_2_O is added (5 mL) and the white solid obtained is filtered, washed several times with H_2_O, and recrystallized from a mixture of ethanol/methanol (1:2) (**8a**, **8c**) or absolute ethanol (**8d**).

*5-Amino-1-(2-hydroxyhexyl)-1H-pyrazole-4-carbohydrazide* **8a**. Yield: 78%. M.p.: 140–142 °C. ^1^H-NMR (400 MHz, CDCl_3_): δ 0.86 (t, J = 5.4 Hz, 3H, CH_3_), 1.15–1.58 (m, 6H, 3CH_2_), 4.56–4.66 (m, 2H, CH_2_N pyraz.), 4.92 (br s, 2H, NH_2_, exchangeable with D_2_O), 5.02 (d, J = 4.4, 1H, OH, exchangeable with D_2_O), 5.13–5.18 (m, 1H, *CH*OH), 6.20 (s, 2H, NH_2_, exchangeable with D_2_O), 7.65 (s, 1H, H-3 pyraz.), 8.97 (br s, 1H, CONH, exchangeable with D_2_O). ^13^C-NMR (101 MHz, CDCl_3_): δ 164.48, 149,03, 138.30, 97.48, 70.38, 53.18, 35.00, 27.54, 22.74, 14.05. Anal calcd. for C_10_H_19_N_5_O_2_. Calcd: %C 49.79, %H 7.94, %N 29.02; found: %C 49.72, %H 7.62, %N 29.30.
*5-Amino-1-(2-hydroxy-2-phenylethyl)-3-methyl-1H-pyrazole-4-carbohydrazide* **8c**. Yield: 85%. M.p.: 224–226 °C. ^1^H-NMR (400 MHz, CDCl_3_): δ 2.24 (s, 3H, CH_3_), 3.83–3.94 (m, 2H, CH_2_N), 4.28 (br s, 2H, NH_2_, exchangeble with D_2_O), 4.84–4.88 (m, 1H, C*H*OH), 5.78 (d, J = 4.4, 1H, OH, exchangeble with D_2_O), 6.03 (s, 2H, NH_2_, exchangeble with D_2_O), 7.23–7.48 (m, 5H, 5Ar), 8.02 (s, 1H, CONH, exchangeble with D_2_O). ^13^C-NMR (101 MHz, CDCl_3_): δ 166.87, 151.19, 150.25, 142.40, 128.56, 128.19, 127.25, 93.34, 73.03, 56.46, 14.93. Anal calcd. for C_13_H_17_N_5_O_2._ Calcd: %C 56.71, %H 6.22, %N 25.44; found: %C 56.94, %H 6.02, %N 25.06.
*5-Amino-1-(2-hydroxy-2-phenylethyl)-1H-pyrazole-3-carbohydrazide* **8d**. Yield: 85%. M.p.: 72–73 °C. ^1^H-NMR (400 MHz, CDCl_3_): δ 3.92–4.08 (m, 2H, CH_2_N), 4.90–4.94 (m, 1H, C*H*OH), 5.23 (br s., 2H, NH_2_, exchangeble with D_2_O), 5.30 (d, J = 4.4, 1H, OH, exchangeble with D_2_O), 5.66 (s, 1H, H-4 pyraz.), 5.91 (s, 2H, NH_2_, exchangeble with D_2_O), 7.11–7.54 (m, 5H, 5Ar), 8.01 (br s, 1H, CONH, exchangebles with D_2_O). ^13^C-NMR (101 MHz, CDCl_3_): δ 162.76, 56.25, 150.62, 145.20, 142.39, 128.56, 128.19, 127.25, 87.47, 72.89. Anal calcd. for C_12_H_15_N_5_O_2_. Calcd: %C 55.16, %H 5.79, %N 26.80; found: %C 55.00, %H 5.49, %N 26.46.

##### Synthesis of Final Compounds **1–4**

To a solution of suitable carbohydrazide **8a–d** (1 mmol) in absolute ethanol (10 mL), the suitable aldehyde **9a–l** (1 mmol), solved in absolute ethanol (2 mL), is added dropwise, then the reaction mixture is heated at reflux for 1–18 h. After cooling to room temperature, the solvent is removed under reduced pressure to obtain yellow/white solids, which are filtered and recrystallized from abs. ethanol (compounds **1**) or diethyl ether (compounds **2–4**).

*(E)-5-Amino-1-(2-hydroxy-2-phenylethyl)-N′-(4-methoxybenzylidene)-1H-pyrazole-4-carbohydrazide* **1a**. Yield: 61%. M.p.: 233–234 °C. ^1^H-NMR (400 MHz, CDCl_3_): δ 3.76 (s, 3H, OCH_3_), 3.84–4.13 (m, 2H, CH_2_N), 4.85–4.88 (m, 1H, *CH*OH), 5.68 (d, J = 4.6 Hz, 1H, OH, exchangeable with D_2_O), 6.12 (s, 2H, NH_2_, exchangeable with D_2_O), 7.25–7.46 (m, 9H, 5Ar + H-2 Ar + H-3 Ar + H-5 Ar + H-6 Ar), 7.86 (s, 1H, H-3 pyraz.), 8.11 (s, 1H, CH=N), 10.98 (s, 1H, CONH, exchangeable with D_2_O). ^13^C-NMR (101 MHz, CDCl_3_): δ 164.49, 161.94, 149.28, 148.53, 142.40, 136.23, 128.80, 128.56, 128.19, 127.25, 126.79, 114.41, 113.80, 97.65, 70.60, 55.72, 55.35. Anal calcd. for C_20_H_21_N_5_O_3_. Calcd: %C 63.31, %H 5.58, %N 18.46; found: %C 63.38, %H 5.62, %N. 18.22.
*(E)-5-Amino-N′-(3,4-dimethoxybenzylidene)-1-(2-hydroxy-2-phenylethyl)-1H-pyrazole-4-carbohydrazide* **1b**. Yield: 32%. M.p.: 89–90 °C. ^1^H-NMR (400 MHz, CDCl_3_): δ 3.74 (s, 3H, OCH_3_), 3.79 (s, 3H, OCH_3_), 3.89–4.10 (m, 2H, CH_2_N), 4.86–4.89 (m, 1H, *CH*OH), 5.67 (d, J = 4.7 Hz, 1H, OH, exchangeable with D_2_O), 6.26 (s, 2H, NH_2_, exchangeable with D_2_O), 6.87–7.42 (m, 8H, 5Ar + H-2 Ar + H-5 Ar + H-6 Ar), 7.85 (s, 1H, H-3 pyraz.), 8.11 (s, 1H, CH=N), 11.02 (s, 1H, CONH, exchangeable with D_2_O). ^13^C-NMR (101 MHz, CDCl_3_): δ 160.95, 151.87, 150.32, 149.28, 144.57, 142.40, 136.31, 128.56, 128.19, 127.99, 127.25, 124.16, 111.32, 109.92, 97.65, 70.76, 55.94, 55.93, 55.72. Anal calcd. for C_21_H_23_N_5_O_4_. Calcd: %C 61.60, %H 5.66, %N 17.10; found: %C 61.25, %H 5.38, %N 17.14.
*(E)-5-Amino-1-(2-hydroxy-2-phenylethyl)-N′-(4-methoxy-3-phenoxybenzylidene)-1H-pyrazole-4-carbohydrazide* **1c**. Yield: 32%. M.p.: 190–191 °C. ^1^H-NMR (400 MHz, CDCl_3_): δ 3.77 (s, 3H, OCH_3_), 3.88–4.07 (m, 2H, CH_2_N), 4.83–4.85 (m, 1H, C*H*OH), 5.66 (d, J = 4.7 Hz, 1H, OH, exchangeable with D_2_O), 6.30 (s, 2H, NH_2_, exchangeable with D_2_O), 6.73–7.49 (m, 13H, 10Ar + H-5 Ar + H-6 Ar + H-2 Ar), 7.77 (s, 1H, H-3 pyraz.), 8.15 (s, 1H, CH=N), 11.01 (s, 1H, CONH, exchangeable with D_2_O). ^13^C NMR (101 MHz, DMSO-*d*_6_): δ 157.89, 152.89, 143.17, 130.41, 128.65, 128.55, 127.92, 126.72, 123.15, 117.08, 72.00, 56.40, 54.46. Anal calcd. for C_26_H_25_N_5_O_4_. Calcd: %C 66.23, %H 5.34, %N 14.85; found: %C 65.86, %H 5.31, %N 14.38.
*(E)-5-Amino-N′-(3-(benzyloxy)-4-methoxybenzylidene)-1-(2-hydroxy-2-phenylethyl)-1H-pyrazole-4-carbohydrazide* **1d**. Yield: 62%. M.p.: 161–163 °C. ^1^H-NMR (400 MHz, CDCl_3_): δ 3.77 (s, 3H, OCH_3_), 3.91–4.09 (m, 2H, CH_2_N), 4.89–4.90 (m, 1H, *CH*OH), 5.10 (s, 2H, CH_2_O), 5.68 (d, J = 4.6 Hz, 1H, OH, exchangeable with D_2_O), 6.39 (s, 2H, NH_2_, exchangeable with D_2_O), 6.92–7.60 (m, 13H, 10Ar + H-5 Ar + H-6 Ar + H-2 Ar), 7.84 (s, 1H, H-3 pyraz.), 8.10 (s, 1H, CH=N), 10.99 (s, 1H, CONH, exchangeable with D_2_O). ^13^C-NMR (101 MHz, CDCl_3_): δ 160.95, 152.83, 149.28, 149.26, 144.57, 142.40, 137.41, 135.44, 128.56, 128.50, 128.19, 128.16, 128.12, 127.71, 127.25, 124.42, 112.85, 112.10, 97.65, 71.88, 71.05, 56.07, 55.72. Anal calcd. for C_27_H_27_N_5_O_4_. Calcd: %C 66.79, %H 5.61, %N 14.42; found: %C 66.66, %H 5.77, %N 14.48.
*(E)-5-Amino-N′-(4-((4-fluorobenzyl)oxy)-3-methoxybenzylidene)-1-(2-hydroxy-2-phenylethyl)-1H-pyrazole-4-carbohydrazide* **1e**. Yield: 75%. M.p.: 136–137 °C. ^1^H-NMR (400 MHz, CDCl_3_): δ 3.78 (s, 3H, OCH_3_), 3.91–4.12 (m, 2H, CH_2_N), 4.91–4.93 (m, 1H, *CH*OH), 5.04 (s, 2H, CH_2_O), 5.69 (d, J = 4.6 Hz, 1H, OH, exchangeable with D_2_O), 6.28 (s, 2H, NH_2_, exchangeable with D_2_O), 7.00–7.56 (m, 12H, 9Ar + H-5 Ar + H-6 Ar + H-2 Ar), 7.86 (s, 1H, H-3 pyraz.), 8.10 (s, 1H, CH=N), 11.04 (s, 1H, CONH, exchangeable with D_2_O). ^13^C NMR (101 MHz, DMSO-*d*_6_): δ 163.59, 161.17, 149.89, 149.63, 143.19, 133.63, 133.60, 130.74, 130.65, 128.67, 128.41, 127.94, 126.73, 115.92, 115.71, 113.81, 72.04, 69.68, 55.94, 54.51, 40.50. Anal calcd. for C_27_H_26_N_5_O_4_F. Calcd: %C 64.40, % H 5.20, %N 13.91; found: %C 64.55, %H 5.37, %N 13.51.
*(E)-5-Amino-N′-(4-((3-fluorobenzyl)oxy)-3-methoxybenzylidene)-1-(2-hydroxy-2-phenylethyl)-1H-pyrazole-4-carbohydrazide* **1f**. Yield: 64%. M.p.: 123–125 °C. ^1^H-NMR (400 MHz, CDCl_3_): δ 3.81 (s, 3H, OCH_3_), 3.90–4.12 (m, 2H, CH_2_N), 4.89–4.91 (m, 1H, *CH*OH), 5.12 (s, 2H, CH_2_O), 5.68 (d, J = 4.6 Hz, 1H, OH, exchangeable with D_2_O), 6.31 (s, 2H, NH_2_, exchangeable with D_2_O), 6.96–7.48 (m, 12H, 9Ar + H-5 Ar + H-6 Ar + H-2 Ar), 7.86 (s, 1H, H-3 pyraz.), 8.10 (s, 1H, CH=N), 11.03 (s, 1H, CONH, exchangeable with D_2_O). ^13^C NMR (101 MHz, DMSO-*d*_6_): δ 163.93, 161.51, 149.90, 149.47, 143.19, 140.40, 140.32, 131.09, 131.01, 128.67, 128.55, 127.94, 126.73, 124.23, 124.20, 115.33, 115.12, 115.00, 114.78, 113.87, 72.03, 69.55, 55.99, 54.50, 40.70, 40.49. Anal calcd. for C_27_H_26_N_5_O_4_F. Calcd: %C 64.40, %H 5.20, %N 13.91; found: %C 64.82, %H 5.16, %N 13.78.
*(E)-5-Amino-N′-(4-((4-chlorobenzyl)oxy)-3-methoxybenzylidene)-1-(2-hydroxy-2-phenylethyl)-1H-pyrazole-4-carbohydrazide* **1g**. Yield: 48%. M.p.: 120–123 °C. ^1^H-NMR (400 MHz, CDCl_3_): δ 3.79 (s, 3H, OCH_3_), 3.85–4.13 (m, 2H, CH_2_N), 4.88–4.91 (m, 1H, *CH*OH), 5.15 (s, 2H, CH_2_O), 5.67 (d, J = 4.4 Hz, 1H, OH, exchangeable with D_2_O), 6.36 (s, 2H, NH_2_, exchangeable with D_2_O), 6.99–7.49 (m, 12H, 9Ar + H-5 Ar + H-6 Ar + H-2 Ar), 7.77 (s, 1H, H-3 pyraz.), 8.12 (s, 1H, CH=N), 11.02 (s, 1H, CONH, exchangeable with D_2_O). ^13^C NMR (101 MHz, DMSO-*d*_6_): δ 149.89, 149.51, 143.19, 136.47, 133.05, 130.19, 129.01, 128.67, 128.49, 127.94, 126.73, 72.03, 69.53, 55.97, 54.50, 40.50. Anal calcd. for C_27_H_26_N_5_O_4_Cl. Calcd: %C 62.37, %H 5.04, %N 13.47; found: %C 62.39, %H 5.28, %N 13.86.
*(E)-5-Amino-N′-(4-(difluoromethoxy)-3-((3-fluorobenzyl)oxy)benzylidene)-1-(2-hydroxy-2-phenylethyl)-1H-pyrazole-4-carbohydrazide* **1h**. Yield: 82%. M.p.: 148–153 °C. ^1^H-NMR (400 MHz, CDCl_3_): δ 3.90–4.12 (m, 2H, CH_2_N), 4.85–4.87 (m, 1H, CHOH), 5.23 (s, 2H, CH_2_O), 5.68 (d, J =4.4, 1H, OH, exchangeable with D_2_O), 6.37 (s, 2H, NH_2_, exchangeable with D_2_O), 7.15 (t, J = 70 Hz, 1H, OCHF_2_), 7.21–7.50 (m, 12H, 9Ar + H-5 Ar + H-6 Ar + H-2 Ar), 7.96 (s, 1H, H-3 pyraz.), 8.21 (s, 1H, CH=N), 11.18 (s, 1H, CONH, exchangeable with D_2_O). ^13^C NMR (101 MHz, DMSO-d_6_): δ 163.98, 161.56, 150.30, 143.17, 141.20, 139.98, 139.91, 133.76, 131.17, 131.08, 128.67, 127.94, 126.74, 123.97, 122.21, 119.88, 117.31, 115.44, 115.23, 114.74, 114.52, 72.03, 69.63, 54.49. Anal calcd. for C_27_H_24_N_5_O_4_F_3_. Calcd: %C 60.11, %H 4.48, %N 12.98; found: %C 60.23, %H 4.60, %N 13.12.
*(E)-5-Amino-N′-(3-((4-chlorobenzyl)oxy)-4-(difluoromethoxy)benzylidene)-1-(2-hydroxy-2-phenylethyl)-1H-pyrazole-4-carbohydrazide* **1i**. Yield: 81%. M.p.: 106–108 °C. ^1^H-NMR (400 MHz, CDCl_3_): δ 3.83–4.14 (m, 2H, CH_2_N), 4.87–4.90 (m, 1H, CHOH), 5.23 (s, 2H, CH_2_O), 5.68 (d, J = 4.4, 1H, OH, exchangeable with D_2_O), 6.32 (s, 2H, NH_2_, exchangeable with D_2_O), 7.13 (t, J = 70 Hz, 1H, OCHF_2_), 7.21–7.53 (m, 12H, 9Ar + H-5 Ar + H-6 Ar + H-2 Ar), 7.94 (s, 1H, H-3 pyraz.), 8.18 (s, 1H, CH=N), 11.18 (s, 1H, CONH, exchangeable with D_2_O). ^13^C NMR (101 MHz, DMSO-d_6_): δ 150.28, 143.17, 141.23, 136.07, 133.68, 133.17, 130.98, 129.98, 129.11, 128.90, 128.67, 127.95, 126.75, 122.07, 119.83, 117.26, 114.69, 112.19, 72.04, 69.66, 54.49, 40.70, 38.25. Anal calcd. for C_27_H_24_N_5_O_4_ClF_2_. Calcd: %C 58.33, %H 4.35, %N 12.60; found: %C 58.10, %H 4.33, %N 12.35.
*(E)-5-Amino-N′-(4-(difluoromethoxy)-3-methoxybenzylidene)-1-(2-hydroxy-2-phenylethyl)-3-methyl-1H-pyrazole-4-carbohydrazide* **2a**. Yield: 46%. M.p.: 193–195 °C. ^1^H-NMR (400 MHz, CDCl_3_): δ 2.31 (s, 3H, CH_3_), 3.65–4.12 (m, 5H, CH_2_N + OCH_3_), 4.83–4.86 (m, 1H, CHOH), 5.70 (d, J = 4.6 Hz, 1H, OH, exchangeable with D_2_O), 6.05 (s, 2H, NH_2_, exchangeable with D_2_O), 7.28 (t, J = 70, 1H, OCHF_2_), 7.37–7.67 (m, 8H, 5 Ar + H-5 Ar + H-6 Ar + H-2 Ar), 8.25 (s, 1H, CH=N), 11.42 (s, 1H, CONH, exchangeable with D_2_O). ^13^C-NMR (101 MHz, CDCl_3_): δ 166.24, 151.94, 150.72, 149.40, 144.53, 144.45, 142.40, 128.30, 128.19, 127.25, 121.90, 118.71, 118.69, 117.87, 115.77, 113.66, 110.53, 93.67, 73.03, 56.45, 56.23, 14.93. Anal calcd. for C_22_H_23_N_5_O_4_F_2_. Calcd: %C 57.51, %H 5.05, %N 15.24; found: %C 57.31, %H 5.26, %N 15.64.
*(E)-5-Amino-N′-(4-(difluoromethoxy)-3-phenoxybenzylidene)-1-(2-hydroxy-2-phenylethyl)-3-methyl-1H-pyrazole-4-carbohydrazide* **2b**. Yield: 25%. M.p.: 203–206 °C. ^1^H-NMR (400 MHz, CDCl_3_): δ 2.51 (s, 3H, CH_3_), 3.71–4.10 (m, 2H, CH_2_N), 4.84–4.85 (m, 1H, CHOH), 5.71 (d, J = 4.6 Hz, 1H, OH, exchangeable with D_2_O), 5.95 (s, 2H, NH_2_, exchangeable with D_2_O), 6.94–7.53 (m, 14H, 10Ar + H-5 Ar + H-6 Ar + H-2 Ar + OCHF_2_), 8.21 (s, 1H, CH=N), 10.32 (s, 1H, CONH, exchangeable with D_2_O). ^13^C-NMR (101 MHz, CDCl_3_): δ 166.24, 156.83, 152.85, 150.72, 147.66, 145.72, 144.77, 142.40, 130.02, 128.19, 127.25, 124.60, 122.31, 122.17, 120.07, 118.73, 117.96, 115.73, 115.71, 115.69, 114.87, 92.84, 74.52, 54.27, 16.24. Anal calcd. for C_27_H_25_N_5_O_4_F_2_. Calcd: %C 62.18, %H 4.83, %N 13.43; found: %C 62.37, %H 4.25, %N 13.34.
*(E)-5-Amino-N′-(3-(benzyloxy)-4-(difluoromethoxy)benzylidene)-1-(2-hydroxy-2-phenylethyl)-3-methyl-1H-pyrazole-4-carbohydrazide* **2c**. Yield: 75%. M.p.: 188–190 °C. ^1^H-NMR (400 MHz, CDCl_3_): δ 2.29 (s, 3H, CH_3_), 3.73–4.15 (m, 2H, CH_2_N), 4.79–4.82 (m, 1H, CHOH), 5.21 (s, 2H, CH_2_O), 5.71 (d, J =4.4, 1H, OH, exchangeable with D_2_O), 6.03 (s, 2H, NH_2_, exchangeable with D_2_O), 7.17 (t, J = 70 Hz, 1H, OCHF_2_), 7.30–7.63 (m, 13H, 10Ar + H-5 Ar + H-6 Ar + H-2 Ar), 8.22 (s, 1H, CH=N), 10.42 (s, 1H, CONH, exchangeable with D_2_O). ^13^C-NMR (101 MHz, CDCl_3_): δ 166.24, 154.48, 150.58, 150.56, 145.94, 144.67, 142.40, 137.49, 128.56, 128.50, 128.22, 128.19, 128.16, 127.71, 127.25, 122.14, 119.79, 117.68, 115.58, 112.92, 112.89, 112.87, 111.68, 95.24, 72.49, 71.24, 54.32, 16.33. Anal calcd. for C_28_H_27_N_5_O_4_F_2_. Calcd: %C 62.80, %H 5.08, %N 13.08; found: %C 62.72, %H 5.18, %N 13.51.
*(E)-5-Amino-N′-(4-(difluoromethoxy)-3-methoxybenzylidene)-1-(2-hydroxyhexyl)-1H-pyrazole-4-carbohydrazide* **3a**. Yield: 52%. M.p.: 164–165 °C. ^1^H-NMR (400 MHz, CDCl_3_): δ 0.85 (s, 3H, CH_3_), 1.07–1.48 (m, 6H, 3CH_2_), 3.64–4.03 (m, 5H, OCH_3_ + CH_2_N), 4.93–4.97 (s, 1H, C*H*OH), 5.67 (d, J = 4.5 Hz, 1H, OH, exchangeable with D_2_O), 6.34 (s, 2H, NH_2_, exchangeable with D_2_O), 7.12 (t, J = 67 Hz, 1H, OCHF_2_), 7.40–7.54 (m, 3H, H-5 Ar + H-6 Ar + H-2 Ar), 7.99 (s, 1H, H-3 pyraz.), 8.18 (s, 1H, CH=N), 11.22 (s, 1H, CONH exchangeable with D_2_O). ^13^C-NMR (101 MHz, CDCl_3_): δ 158.95, 151.94, 149.02, 144.53, 135.49, 128.30, 121.90, 118.69, 115.77, 113.66, 110.53, 97.46, 70.38, 56.23, 55.36, 32.62, 27.54, 22.74, 16.41. Anal calcd. for C_19_H_25_N_5_O_4_F_2_. Calcd: %C 53.64, %H 5.92, %N 16.46; found: %C 53.75, %H 5.81, %N 16.36.
*(E)-5-Amino-N′-(4-(difluoromethoxy)-3-phenoxybenzylidene)-1-(2-hydroxyhexyl)-1H-pyrazole-4-carbohydrazide* **3b**. Yield: 73%. M.p.: 140–141 °C. ^1^H-NMR (400 MHz, CDCl_3_): δ 0.86 (t, 3H, CH_3_), 1.14–1.52 (m, 6H, 3CH_2_), 3.65–3.97 (m, 2H, CH_2_N), 4.92–4.94 (m, 1H, C*H*OH), 6.01 (d, J = 4.4 Hz, 1H, OH, exchangeable with D_2_O), 6.29 (s, 2H, NH_2_, exchangeable with D_2_O), 6.83 (t, J = 67 Hz, 1H, OCHF_2_), 7.08–7.63 (m, 8H, 5 Ar + H-5 Ar + H-6 Ar + H-2 Ar), 7.79 (s, 1H, H-3 pyraz.), 8.15 (s, 1H, CH=N), 11.18 (s, 1H, CONH, exchangeable with D_2_O). ^13^C-NMR (101 MHz, CDCl_3_): δ 165.06, 156.83, 149.02, 147.65, 145.13, 145.08, 144.73, 134.57, 130.02, 128.30, 124.60, 122.31, 118.73, 117.87, 115.76, 114.87, 113.66, 97.46, 70.38, 53.17, 32.77, 27.54, 24.62, 18.06. Anal calcd. for C_24_H_27_N_5_O_4_F_2_. Calcd: %C 59.13, %H 5.58, %N 14.37; found: %C 59.59, %H 5.46, %N 14.69.
*(E)-5-Amino-N′-(3-(benzyloxy)-4-(difluoromethoxy)benzylidene)-1-(2-hydroxyhexyl)-1H-pyrazole-4-carbohydrazide* **3c**. Yield: 52%. M.p.: 129–131 °C. ^1^H-NMR (400 MHz, CDCl_3_): δ 0.84 (t, J = 5.6 Hz, 3H, CH_3_), 1.18–1.65 (m, 6H, 3CH_2_), 3.65–3.99 (m, 2H, CH_2_N), 4.85–4.86 (m, 1H, *CH*OH), 5.23 (s, 2H, CH_2_O), 6.03 (d, J = 4.4 Hz, 1H, OH, exchangeable with D_2_O), 6.35 (s, 2H, NH_2_, exchangeable with D_2_O), 7.01 (t, J = 67 Hz, 1H, OCHF_2_), 7.15–7.63 (m, 8H, 5 Ar + H-5 Ar + H-6 Ar + H-2 Ar), 7.97 (s, 1H, H-3 pyraz.), 8.10 (s, 1H, CH=N), 11.22 (s, 1H, CONH, exchangeable with D_2_O). ^13^C-NMR (101 MHz, CDCl_3_): δ 162.95, 150.55, 149.02, 144.54, 137.49, 135.36, 128.22, 128.16, 127.71, 122.14, 118.44, 118.42, 116.84, 114.74, 112.63, 111.68, 95.84, 71.24, 70.38, 53.17, 31.77, 27.54, 24.31, 18.41. Anal calcd. for C_25_H_29_N_5_O_4_F_2_. Calcd: %C 59.87, %H 5.83, %N 13.96; found: %C 59.59, %H 5.77, %N 14.10.
*(E)-5-Amino-N′-(4-(difluoromethoxy)-3-methoxybenzylidene)-1-(2-hydroxy-2-phenylethyl)-1H-pyrazole-3-carbohydrazide* **4a**. Yield: 54%. M.p.: 196–197 °C. ^1^H-NMR (400 MHz, CDCl_3_): δ 3.86–3.94 (m, 2H, CH_2_N), 3.97 (s, 3H, OCH_3_), 4.52 (d, J = 4.4 Hz, 1H, OH, exchangeable with D_2_O), 5.10–5.13 (m, 1H, C*H*OH), 5.69 (s, 2H, NH_2_, exchangeable with D_2_O), 5.74 (s, 1H, H-4 pyraz.), 7.04 (t, J = 70 Hz, 1H, OCHF2), 7.10–7.51 (m, 8H, 5Ar + H-5 Ar + H-6 Ar + H-2 Ar), 8.55 (s, 1H, CH=N), 11.78 (s, 1H, CONH, exchangeable with D_2_O). ^13^C NMR (101 MHz, DMSO-*d*_6_): δ 166.71, 165.11, 150.37, 150.03, 137.50, 136.45, 97.39, 95.92, 69.85, 53.18, 34.39, 27.68, 22.69, 14.51. Anal calcd. for C_21_H_21_N_5_O_4_F_2_. Calcd: %C 56.60, %H 4.70, %N 15.72; found: %C 56.69, %H 4.42, %N 15.69.
*(E)-5-Amino-N′-(4-(difluoromethoxy)-3-phenoxybenzylidene)-1-(2-hydroxy-2-phenylethyl)-1H-pyrazole-3-carbohydrazide* **4b**. Yield: 59%. M.p.: 195–196 °C. ^1^H-NMR (400 MHz, CDCl_3_): δ 3.92–4.08 (m, 2H, CH_2_N), 4.96–4.98 (m, 1H, C*H*OH), 5.33 (d, J = 4.4 Hz, 1H, OH, exchangeable with D_2_O), 5.43 (s, 2H, NH_2_, exchangeable with D_2_O), 5.75 (s, 1H, H-4 pyraz.), 6.92–7.50 (m, 14H, 10Ar + H-5 Ar + H-6 Ar + H-2 Ar + OCHF_2_), 8.40 (s, 1H, CH=N), 11.41 (s, 1H, CONH, exchangeable with D_2_O). ^13^C-NMR (101 MHz, CDCl_3_): δ 162.64, 156.83, 151.88, 150.61, 145.08, 144.66, 142.39, 140.61, 130.02, 128.56, 128.32, 128.19, 127.25, 124.60, 122.31, 121.36, 119.25, 118.73, 117.15, 115.73, 115.70, 115.68, 114.87, 87.65, 71.77, 57.42. Anal calcd. for C_26_H_23_N_5_O_4_F_2_. Calcd: %C 61.53, %H 4.57, %N 13.80; found: %C 61.63, %H 4.95, %N 13.65.
*(E)-5-Amino-N′-(3-(benzyloxy)-4-(difluoromethoxy)benzylidene)-1-(2-hydroxy-2-phenylethyl)-1H-pyrazole-3-carbohydrazide* **4c**. Yield: 15%. M.p.: 136–137 °C. ^1^H-NMR (400 MHz, CDCl_3_): δ 4.00–4.14 (m, 2H, CH_2_N), 4.98–5.01 (m, 1H, C*H*OH), 5.17 (s, 2H, CH_2_O), 5.36 (d, J = 4.4 Hz, 1H, OH, exchangeable with D_2_O), 5.75 (s, 2H, NH_2_, exchangeable with D_2_O), 5.81 (s, 1H, H-4 pyraz.), 7.02 (t, J = 70 Hz, 1H, OCHF_2_), 7.22–7.53 (m, 13H, 10Ar + H-5 Ar + H-6 Ar + H-2 Ar), 8.43 (s, 1H, CH=N), 11.44 (s, 1H, CONH, exchangeable with D_2_O). ^13^C-NMR (101 MHz, CDCl_3_): δ 162.95, 153.71, 153.70, 150.61, 144.49, 144.43, 144.36, 142.39, 140.74, 137.49, 128.50, 128.19, 128.18, 128.16, 127.71, 127.25, 122.14, 120.67, 118.57, 116.46, 112.90, 112.88, 111.68, 86.98, 73.64, 71.24, 57.37. Anal calcd. for C_27_H_25_N_5_O_4_F_2_. Calcd: %C 62.18, %H 4.83, %N 13.43; found: %C 62.15, %H 4.40, %N 13.01.

### 4.2. In Vitro Antioxidant Activity (DPPH Assay) 

The antioxidant activity was measured using the DPPH antioxidant assay. The assay is based on the bleaching rate of the stable radical DPPH [56]. Briefly, ca 3 mg of single compound was dissolved with methanol, then 0.1 mL of this solution was mixed with 3.9 mL of DPPH methanol solution (65 µM). Absorbance was measured at 517 nm after reacting for 30 min in the dark. Linear calibration curve was obtained using Trolox standards (range between 20 to 200 mg/L, R2 = 0.9988). The result was calculated as Trolox equivalents in mg/L and the percentage of antioxidant activity (AA%) was calculated from the ratio of decreasing absorbance of sample solution (A0 − As) to absorbance of blank DPPH solution (A0), as expressed in Equation (1) [57].

AA% = [(A0 − As)/A0] × 100(1)

### 4.3. Human Platelet Assays

#### 4.3.1. Material

2′,7′-Dichlorofluorescein diacetate (DCFH-DA) and thrombin were purchased from Sigma-Aldrich (St. Louis, MI, USA)/Merck Millipore (Burlington, VT, USA).

#### 4.3.2. Blood Collection and Preparative Procedures

Washed platelets were prepared from freshly drawn venous blood obtained from healthy volunteers at the Centro Trasfusionale, Ospedale San Martino in Genoa. Donors declare that they have not taken any drugs known to interfere with platelet function for at least two weeks prior to blood collection and gave their informed consent. Blood was collected into anticoagulant solution containing 130 mM aqueous trisodium citrate (9:1). Whole blood is centrifuged at 100× *g* for 20 min to obtain platelet-rich plasma (PRP) that is further centrifuged at 1100× *g* for 15 min. The obtained pellet is washed once with an acidic solution containing 75 mM trisodium citrate, 42 mM citric acid, and 136 mM glucose (pH 5.2) and then resuspended in a pH 7.4 Hepes buffer solution (145 mM NaCl, 5 mM KCl, 1 mM MgSO_4_, 10 mM glucose, and 10 mM HEPES).

IC_50_ values reported represent the molar concentration of a compound required to inhibit 50% of the maximal effect induced by thrombin. The percentage of inhibition is calculated by comparing the inhibition of the maximal effect measured in the presence of the test compound with that measured in a control sample containing saline, under the same experimental conditions.

#### 4.3.3. ROS Assay

ROS production was measured by the ROS-sensitive probe DCFH-DA, which, upon oxidation, forms the fluorescent compound DCF. The DCF formed by ROS is trapped inside the cells, allowing for the quantification of ROS levels [58]. Washed platelets (1.0 × 10^8^/mL) were preincubated with either saline or test compounds for 15 min at 37 °C. Platelets were then stimulated by 0.1 U/mL thrombin for 15 min at 37 °C. After stimulation, the incubation was stopped by cooling in an ice bath; the samples were immediately analyzed using a flow cytometer (Merck Millipore Bioscience Guava easyCyte flow cytometer). IC_50_ values were calculated as described above.

#### 4.3.4. Platelet Aggregation

Born’s method [59] is a well-established technique for studying platelet aggregation in response to various stimuli and it provides valuable information about the ability of compounds to modulate platelet function. 

Washed platelets (3.0 × 10^8^/mL) were preincubated for 3 min at 37 °C with either saline or test compounds and then stimulated by 0.1 U/mL thrombin. In a BioData Aggregometer (Bio-Data Corporation, Horsham, PA, USA), platelet aggregation is quantified by measuring the light transmission over 6 min at 37 °C. IC_50_ values were calculated as above reported.

### 4.4. Cell Growth Inibitory activity

**1c**, **1d**, **1f**, and **1g** were tested at a fixed concentration of 10 μM against three different breast cancer cell lines (namely, MCF7, MDA-MB231, SK-BR3) using Cisplatin (Cis-Pt) as reference compound. Cisplatin was kindly provided by the pharmacy (UFA-Unità Farmaci Antiblastici) of the IRCCS Ospedale Policlinico San Martino.

#### MTT Assay

To perform MTT assay, SK-BR3 (breast adenocarcinoma, Biologic Bank and Cell Factory, IRCCS Policlinico San Martino, Genoa, Italy), MCF-7 (breast adenocarcinoma, Biologic Bank and Cell Factory, IRCCS Policlinico San Martino, Genoa, Italy), and MDA-MB231 (breast adenocarcinoma, Biologic Bank and Cell Factory, IRCCS Policlinico San Martino, Genoa, Italy) cell lines were cultured in Dulbecco’s Modified Eagle Medium (DMEM) added to 10% Fetal bovine serum (FBS), 2 mM Glutamine, and 1% penstrep. Reagents were acquired from EuroClone (Milan, Italy) and incubated in a humidified environment at 37 °C with 5% CO_2_. All chemical compounds (**1c**, **1d**, **1f**, and **1g** and Cisplatin, used as reference compound) were dissolved in DMSO to give a 10 mM stock solution. Then, after an intermediate dilution in growth medium, they were added to the cultured cells at a final working concentration of 10 μM and incubated for 48 h. At the end of the incubation, 30 μL of MTT (3-(4,5-dimethyl-2-thiazolyl)-2,5-diphenyl-2H-tetrazolium bromide) at a concentration of 2 mg/mL in PBS, were added in each well and incubated 4 h. Finally, the supernatants were removed and 100 μL/well of DMSO were added to each well to dissolve the Formazan precipitates. After 20 min, the results were read at λ = 570 nm. Results are expressed as a percentage of the control samples, where cells have been treated with the same amount of DMSO but without any chemical compound. The assay was repeated three times, and a single compound was tested six times. Means and standard deviations were calculated.

The GI_50_ values were calculated based on single concentration-response curves. Each experiment was repeated three times.

## Figures and Tables

**Table 1:** Different decoration of novel compounds **1–4**.

Compounds	R′	R″
**1a**	//	CH_3_
**1b**	CH_3_	CH_3_
**1c**	C_6_H_5_	CH_3_
**1d**	CH_2_C_6_H_5_	CH_3_
**1e**	CH_3_	CH_2_C_6_H_4_-*p*F
**1f**	CH_3_	CH_2_C_6_H_4_-*m*F
**1g**	CH_3_	CH_2_C_6_H_4_-*p*Cl
**1h**	CH_2_C_6_H_4_-*m*F	CHF_2_
**1i**	CH_2_C_6_H_4_-*p*Cl	CHF_2_
**2a**	CH_3_	CHF_2_
**2b**	C_6_H_5_	CHF_2_
**2c**	CH_2_C_6_H_5_	CHF_2_
**3a**	CH_3_	CHF_2_
**3b**	C_6_H_5_	CHF_2_
**3c**	CH_2_C_6_H_5_	CHF_2_
**4a**	CH_3_	CHF_2_
**4b**	C_6_H_5_	CHF_2_
**4c**	CH_2_C_6_H_5_	CHF_2_

**Table 2:** Evaluation of antioxidant activity percent (AA%) using DPPH assay.

Compound	A (517 nm) ^a^	DPPH% ^b^	AA% ^b^
**1a**	0.917	99.48 ± 0.31	0.30 ± 0.31
**1b**	0.909	98.93 ± 0.15	1.17 ± 0.15
**1c**	0.917	99.59 ± 0.08	0.36 ± 0.08
**1d**	0.881	95.56 ± 0.31	4.22 ± 0.31
**1e**	0.910	98.83 ± 0.15	1.07 ± 0.15
**1f**	0.912	99.04 ± 0.15	0.85 ± 0.15
**1g**	0.909	98.72 ± 0.15	1.17 ± 0.15
**1h**	0.869	94.59 ± 0.15	5.52 ± 0.15
**1i**	0.864	93.93 ± 0.08	6.12 ± 0.08
**2a**	0.900	97.85 ± 0.08	2.21 ± 0.08
**2b**	0.867	94.48 ± 0.38	5.79 ± 0.38
**2c**	0.872	94.91 ± 0.23	5.25 ± 0.23
**3a**	0.914	98.93 ± 0.54	0.68 ± 0.54
**3b**	0.915	99.37 ± 0.08	0.58 ± 0.08
**3c**	0.865	94.37 ± 0.46	5.96 ± 0.46
**4a**	0.873	94.69 ± 0.31	5.09 ± 0.31
**4b**	0.666	72.08 ± 0.38	27.65 ± 0.38
**4c**	0.778	84.47 ± 0.08	15.47 ± 0.08
**DPPH**	0.920	-	-

^a^ Absorbance. ^b^ Mean value ± standard deviation (SD) of two independent experiments (*n* = 2).

**Table 3:** Inhibitory effect of new APs **1–4** and reference compounds **ASA** (acetylsalicylic acid) and **NAC** (N-acetylcysteine) on platelet aggregation and ROS production on human platelets expressed as IC_50_ (μM) values.

	IC_50_ (μM) ^a^	
Compound	Aggregation Inhibition	ROS Production Inhibition
**1a**	560 ± 27	473 ± 43
**1b**	137 ± 5	143 ± 12
**1c**	483 ± 12	451 ± 42
**1d**	851 ± 17	923 ± 62
**1e**	953 ± 18	960 ± 32
**1f**	>1000	>1000
**1g**	914 ± 22	902 ± 43
**1h**	187 ± 33	236 ± 47
**1i**	190 ± 43	240 ± 53
**2a**	221 ± 26	198 ± 26
**2b**	334 ± 22	302 ± 36
**2c**	>1000	>1000
**3a**	130 ± 18	143 ± 13
**3b**	113 ± 3	128 ± 5
**3c**	116 ± 3	139 ± 12
**4a**	146 ± 24	186 ± 18
**4b**	178 ± 27	145 ± 28
**4c**	169 ± 28	205 ± 24
**ASA**	438 ± 18	n.d.
**NAC**	n.d.	872 ± 26

^a^ Reported data are the mean +/− SD obtained in six different experiments each performed in duplicate. n.d.: not determined.

**Table 4:** Cell growth percent values of most active 5APS **1–4** on different cancer cell lines at 10 μM concentration. For each compound, only cell lines with a growth percent values ≤ 50% are indicated.

Compound	Cancer Cell Lines		Cell Growth Percent (%)
**1c**	Leukemia	RPMI-8226 SR	48.0 44.43
	Colon Cancer	HCT-15 KM12	49.2 50.97
	Breast Cancer	T-47D	33.11
**1d**	Leukemia	CCRF-CEM MOLT-4 K-562 SR	34.92 43.36 50.96 40.25
	Colon Cancer	KM12	44.11
	Breast Cancer	T-47D	25.19
**1e**	Renal Cancer	CAKI-1	32.41
**1f**	Leukemia	CCRF-CEM RPMI-8226	43.46 50.80
	Breast Cancer	T-47D	35.88
**1g**	Colon Cancer	HCT-116	49.35
	Ovarian Cancer	OVCAR-3	45.03
	Breast Cancer	HS 578t	43.21
**3a**	Leukemia	K-562 MOLT-4 RPMI-8226 SR	38.64 37.84 45.20 40.35
	Melanoma	SK-MEL-5	50.43
	Breast Cancer	T-47D	44.38
**3c**	Leukemia	CCRF-CEM K-562 MOLT-4 RPMI-8226 SR	45.70 29.16 35.03 46.77 41.04
	Prostate Cancer	PC-3	48.28

**Table 5:** Cell growth percent values of **1c**, **1d**, **1f,** and **1g** and Cisplatin, used as reference compound, on three breast cancer cell lines at 10 μM concentration. Data are mean values for three separate experiments. Variation among triplicate samples is less than 10%. For **1g**, GI_50_ values (μM) were also reported.

	1c	1d	1f	1g	Cis-Pt
Breast cancer cell line					
*MCF7*	>80	>80	>80	52.14%	72.74%
*MDA-MB231*	>80	>80	>80	55.0%	72.74%
*SK-BR3*	>80	>80	65.37%	44.78% (GI_50_ = 14.4 ± 0.3 μM)	70.59% (GI_50_ = 25.7 ± 3.3 μM)

## Data Availability

The data presented in this study are available in article and Supplementary Materials.

## Supplementary Materials

The following supporting information can be downloaded at: https://www.mdpi.com/article/10.3390/molecules29102298/s1, Table S1. Predicted pharmacokinetics and drug-like properties of compounds **1a-i**. Table S2. Predicted pharmacokinetics and drug-like properties of compounds **2a–c**, **3a–c**, **4a–c**. Table S3. Predicted toxicity of compounds **1a–i, 2a–c**, **3a–c**, **4a–c**. Figures S1–S42: ^1^H NMR (400 MHz) and ^13^C NMR (101 MHz) of compounds **8a**,**8c**,**8d** and **1–4**.
