# Supplementary material for: Investigations of Antioxidant and Anti-Cancer Activities of 5-Aminopyrazole Derivatives
Source: Molecules. 2024 May 14;29(10):2298. doi: 10.3390/molecules29102298 (PMC11124527; doi:10.3390/molecules29102298)

Investigations of antioxidant and anti-cancer activities of 5-aminopyrazole derivatives

Federica Rapetti[1], Andrea Spallarossa[1], Eleonora Russo[1], Debora Caviglia [1], Carla Villa[1], Bruno Tasso[1], Maria Grazia Signorello[2], Camillo Rosano[3], Erika Iervasi[3], Marco Ponassi[3] and Chiara Brullo[1*]

1 Department of Pharmacy (DIFAR), University of Genoa, Viale Benedetto XV, 3, 16132 Genoa, Italy; federica.rapetti@unige.it (F.R.); andrea.spallarossa@unige.it (A.S.), bruno.tasso@unige.it (B.T.); eleonora.russo@unige.it, debora.caviglia@edu.unige.it (D.C.), carla.villa@unige.it (C.V.).

2 Department of Pharmacy, Biochemistry Laboratory, University of Genoa, Viale Benedetto XV 3, I-16132 Genova, Italy; mariagrazia.signorello@unige.it (MGS);

3 IRCCS Ospedale Policlinico San Martino, Proteomics and Mass Spectrometry Unit, L.go. R. Benzi, 10, 16132 Genova, Italy; E-mail: camillo.rosano@hsanmartino.it (CR); marco.ponassi@hsanmartino.it (MP); iervasierika@gmail.com (EI).

* Correspondence: chiara.brullo@unige.it

Supporting Material:

Table S1. Predicted pharmacokinetics and drug-like properties of compounds **1a-i**
Table S2. Predicted pharmacokinetics and drug-like properties of compounds **2a-c, 3a-c, 4a-c.**
Table S3. Predicted toxicity of compounds **1a-I, 2a-c, 3a-c, 4a-c.**
Figure S1:^{1}H NMR (400 MHz) of compound **8a**, **Figure S2**: ^{13}C NMR (101 MHz) of compound **8a**
Figure S3:^{1}H NMR (400 MHz) of compound **8c**, **Figure S4**: ^{13}C NMR (101 MHz) of compound **8c**
Figure S5:^{1}H NMR (400 MHz) of compound **8d**, **Figure S6**: ^{13}C NMR (101 MHz) of compound **8d**
Figure S7:^{1}H NMR (400 MHz) of compound **1a**, **Figure S8**: ^{13}C NMR (101 MHz) of compound **1a**
Figure S9:^{1}H NMR (400 MHz) of compound **1b**, **Figure S10**: ^{13}C NMR (101 MHz) of compound **1b**
Figure S11:^{1}H NMR (400 MHz) of compound **1c**, **Figure S12**: ^{13}C NMR (101 MHz) of compound **1c**
Figure S13:^{1}H NMR (400 MHz) of compound **1d**, **Figure S14**: ^{13}C NMR (101 MHz) of compound **1d**
Figure S15:^{1}H NMR (400 MHz) of compound **1e**, **Figure S16**: ^{13}C NMR (101 MHz) of compound **1e**
Figure S17:^{1}H NMR (400 MHz) of compound **1f**, **Figure S18**: ^{13}C NMR (101 MHz) of compound **1f**
Figure S19:^{1}H NMR (400 MHz) of compound **1g**, **Figure S20**: ^{13}C NMR (101 MHz) of compound **1g**
Figure S21:^{1}H NMR (400 MHz) of compound **1h**, **Figure S22:** ^{13}C NMR (101 MHz) of compound **1h**
Figure S23:^{1}H NMR (400 MHz) of compound **1i**, **Figure S24**: ^{13}C NMR (101 MHz) of compound **1i**
Figure S25:^{1}H NMR (400 MHz) of compound **2a**, **Figure S26**: ^{13}C NMR (101 MHz) of compound **2a**
Figure S27:^{1}H NMR (400 MHz) of compound **2b**, **Figure S28**: ^{13}C NMR (101 MHz) of compound **2b**
Figure S29:^{1}H NMR (400 MHz) of compound **2c**, **Figure S30**: ^{13}C NMR (100 MHz) of compound **2c**
Figure S31:^{1}H NMR (400 MHz) of compound **3a**, **Figure S32**: ^{13}C NMR (101 MHz) of compound **3a**
Figure S33:^{1}H NMR (400 MHz) of compound **3b**, **Figure S34**: ^{13}C NMR (101 MHz) of compound **3b**
Figure S35:^{1}H NMR (400 MHz) of compound **3c**, **Figure S36**: ^{13}C NMR (101 MHz) of compound **3c**
Figure S37:^{1}H NMR (400 MHz) of compound **4a**, **Figure S38**: ^{13}C NMR (101 MHz) of compound **4a**
Figure S39:^{1}H NMR (400 MHz) of compound **4b**, **Figure S40**: ^{13}C NMR (101 MHz) of compound **4b**
Figure S41:^{1}H NMR (400 MHz) of compound **4c**, **Figure S42**: ^{13}C NMR (101 MHz) of compound **4c**

Table S1. Predicted pharmacokinetics and drug-like properties of compounds **1a-i**

	**1a**	**1b**	**1c**	**1d**	**1e**	**1f**	**1g**	**1h**	**1i**
**Physicochemical Prop.**									
MW (g/mol)	379.41	409.44	471.51	485.53	503.52	503.52	519.98	539.51	555.96
Fraction Csp^3	15	19	12	15	15	15	15	15	15
Rotatable bonds	8	9	10	11	11	11	11	12	12
H-bond acceptors	5	6	6	6	7	7	6	9	8
H-bond donors	3	3	3	3	3	3	3	3	3
TPSA[a] (Å^2)	114.76	123.99	123.99	123.99	123.99	123.99	123.99	123.99	123.99
**Lipophilicity**									
LogP[b]	2.1	2.07	3.62	3.56	3.66	3.66	4.19	4.61	5.14
**Water solubility**									
Solubility (mg/ml)[c]	13.9	12.7	0.636	0.702	0.502	0.502	0.19	0.0996	0.0376
Solubility class	Soluble	Soluble	Mod. soluble	Mod. soluble	Mod. soluble	Mod. soluble	Mod. soluble	Mod. soluble	Poorly soluble
**Pharmacokinetics**									
GI absorption	high	high	high	high	high	high	high	low	low
BBB permeant	no	no	no	no	no	no	no	no	no
Pgp substrate	yes	yes	no	yes	yes	yes	no	no	no
CYP1A2 inhibitor	no	no	no	no	no	no	no	no	no
CYP2C19 inhibitor	no	no	yes	yes	yes	yes	yes	yes	yes
CYP2C9 inhibitor	no	no	yes	yes	yes	yes	yes	yes	yes
CYP2D6 inhibitor	no	no	yes	yes	yes	yes	yes	yes	yes
CYP3A4 inhibitor	no	no	no	yes	yes	yes	yes	yes	yes
**Druglikeness**									
Lipinski violations	0	0	0	0	1	1	1	1	1
**Medicinal chemistry**									
PAINS alerts	0	0	0	0	0	0	0	0	
Brenk alerts	1 (imine)	1 (imine)	1 (imine)	1 (imine)	1 (imine)	1 (imine)	1 (imine)	1 (imine)	1 (imine)

Mod.: Moderately. [a] Topological Polar Surface Area. [b] Predicted according to XLOGP3 program. [c] Values predicted by ESOL method [Delaney, J.S. ESOL: Estimating Aqueous Solubility Directly from Molecular Structure. *J Chem Inf Model* **2004**, *44*, 1000-1005. DOI: 10.1021/ci034243x.]

Table S2. Predicted pharmacokinetics and drug-like properties of compounds **2a-c, 3a-c, 4a-c.**

	**2a**	**2b**	**2c**	**3a**	**3b**	**3c**	**4a**	**4b**	**4c**
**Physicochemical Prop.**									
MW (g/mol)	459.45	521.52	535.54	425.43	487.50	501.53	445.42	507.49	521.52
Fraction Csp^3	23	15	18	42	29	32	19	12	15
Rotatable bonds	10	11	12	12	13	14	10	11	12
H-bond acceptors	8	8	8	8	8	8	8	8	8
H-bond donors	3	3	3	3	3	3	3	3	3
TPSA[a] ($Å^2$)	114.76	123.99	123.99	123.99	123.99	123.99	123.99	123.99	123.99
**Lipophilicity**									
LogP[b]	2.56	4.98	4.91	3.35	4.9	4.84	2.80	4.36	4.29
**Water solubility**									
Solubility (mg/ml)[c]	4.36	0.0609	0.0677	3.64	0.173	0.189	3.55	0.173	0.193
Solubility class	Mod. soluble	Mod. soluble	Mod. soluble	Mod. soluble	Mod. soluble	Mod. soluble	Mod. soluble	Mod. soluble	Mod. soluble
**Pharmacokinetics**									
GI absorption	high	low	low	high	low	low	high	low	low
BBB permeant	no	no	no	no	no	no	no	no	no
Pgp substrate	yes	no	no	yes	no	no	yes	no	no
CYP1A2 inhibitor	no	no	no	no	no	no	no	no	no
CYP2C19 inhibitor	yes	yes	yes	yes	yes	yes	no	yes	yes
CYP2C9 inhibitor	no	yes	yes	no	yes	yes	yes	yes	yes
CYP2D6 inhibitor	no	yes	yes	no	yes	yes	no	yes	yes
CYP3A4 inhibitor	no	yes	yes	yes	yes	yes	no	no	yes
**Druglikeness**									
Lipinski violations	0	1	1	0	0	1	0	1	1
**Medicinal chemistry**									
PAINS alerts	0	0	0	0	0	0	0	0	
Brenk alerts	1 (imine)	1 (imine)	1 (imine)	1 (imine)	1 (imine)	1 (imine)	1 (imine)	1 (imine)	1 (imine)

Mod.: Moderately . [a] Topological Polar Surface Area. [b] Predicted according to XLOGP3 program. [c] Values predicted by ESOL method [Delaney, J.S. ESOL: Estimating Aqueous Solubility Directly from Molecular Structure. *J Chem Inf Model* **2004**, *44*, 1000-1005. DOI: 10.1021/ci034243x.]

Table S3. Predicted toxicological profile of compounds **1a-i**, **2a-c**, **3a-c**, **4a-c**.

Compound	Predicted acute rodent toxicity LD_{50}	Predicted Toxicity class [a]	Predicted organ toxicity [b]	Toxicological endpoints [b]	Toxicity targets
**1a**	4540 mg/kg	5	Neurotoxicity Respiratory toxicity	none	none
**1b**	4540 mg/kg	5	Respiratory toxicity	Immunotoxicity	none
**1c**	4540 mg/kg	5	Respiratory toxicity	Immunotoxicity	none
**1d**	4540 mg/kg	5	Respiratory toxicity	Immunotoxicity	none
**1e**	4540 mg/kg	5	Neurotoxicity Respiratory toxicity	Immunotoxicity	none
**1f**	4540 mg/kg	5	Neurotoxicity Respiratory toxicity	Immunotoxicity	none
**1g**	1000 mg/kg	4	Neurotoxicity Respiratory toxicity	Immunotoxicity	none
**1h**	4540 mg/kg	5	Neurotoxicity Respiratory toxicity	Immunotoxicity	none
**1i**	1000 mg/kg	4	Neurotoxicity Respiratory toxicity	Immunotoxicity	none
**2a**	1000 mg/kg	4	Neurotoxicity Respiratory toxicity	Immunotoxicity	none
**2b**	4920 mg/kg	5	Neurotoxicity Respiratory toxicity	Immunotoxicity	none
**2c**	4540 mg/kg	5	Neurotoxicity Respiratory toxicity	none	none
**3a**	4540 mg/kg	5	Respiratory toxicity	none	none
**3b**	4920 mg/kg	5	Respiratory toxicity	none	none
**3c**	4920 mg/kg	5	Respiratory toxicity	none	none
**4a**	4920 mg/kg	5	Neurotoxicity Respiratory toxicity	Immunotoxicity	none
**4b**	4920 mg/kg	5	Neurotoxicity Respiratory toxicity	Immunotoxicity	none
**4c**	6000 mg/kg	6	Neurotoxicity Respiratory toxicity	Immunotoxicity	none

[a]Prediction accurancy 54.26%; [b] probability ≥ 70%;

Figure S1: ¹H NMR (400 MHz) of compound **8a**

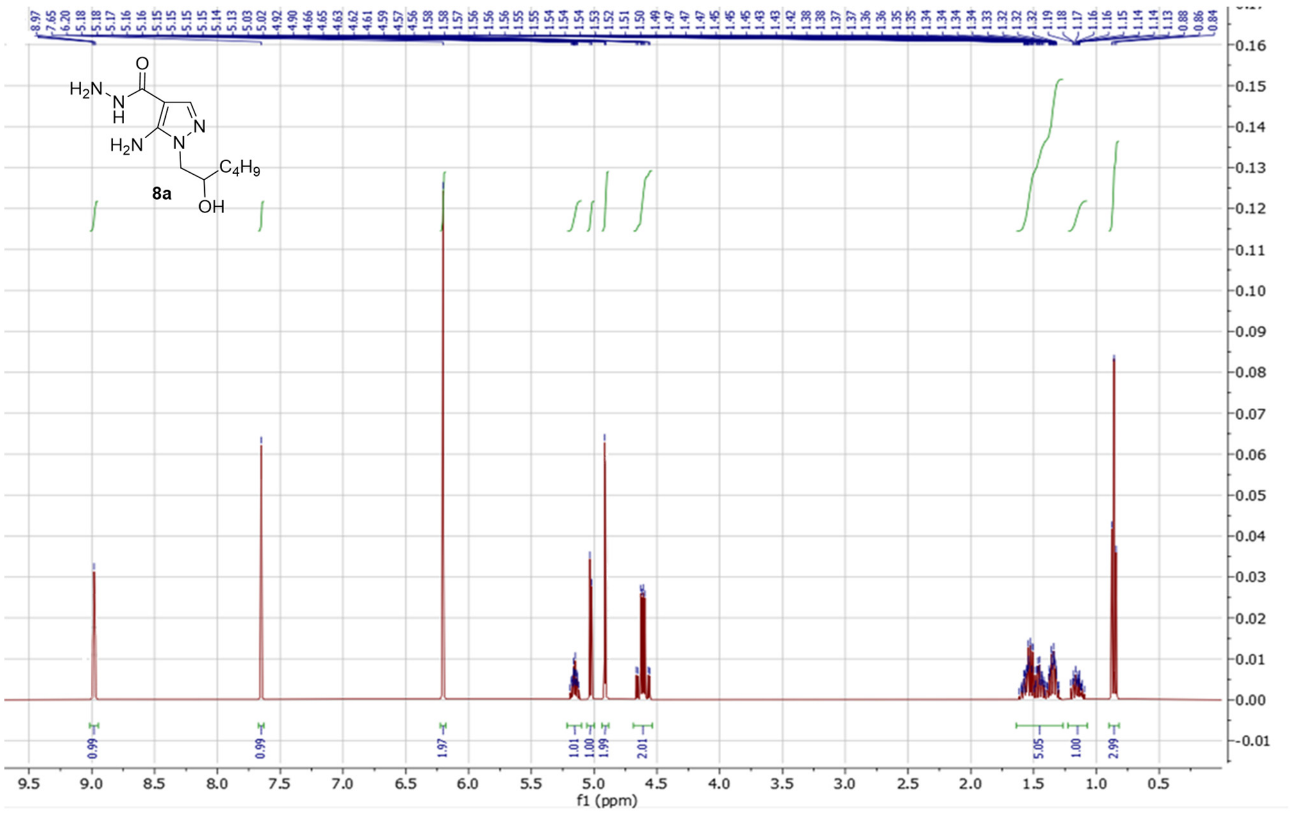

Figure S2: ¹³C NMR (101 MHz) of compound **8a**

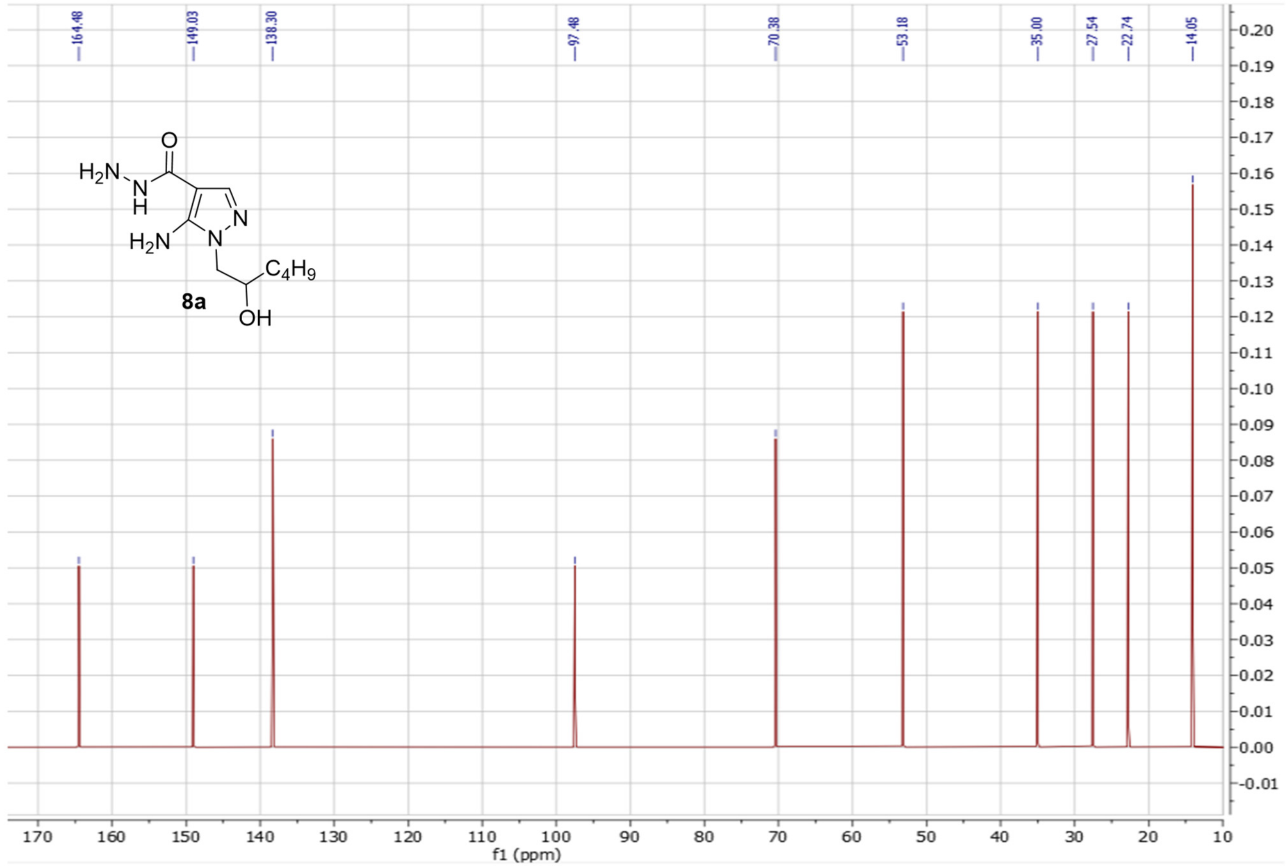

Figure S3:^{1}H NMR (400 MHz) of compound **8c**

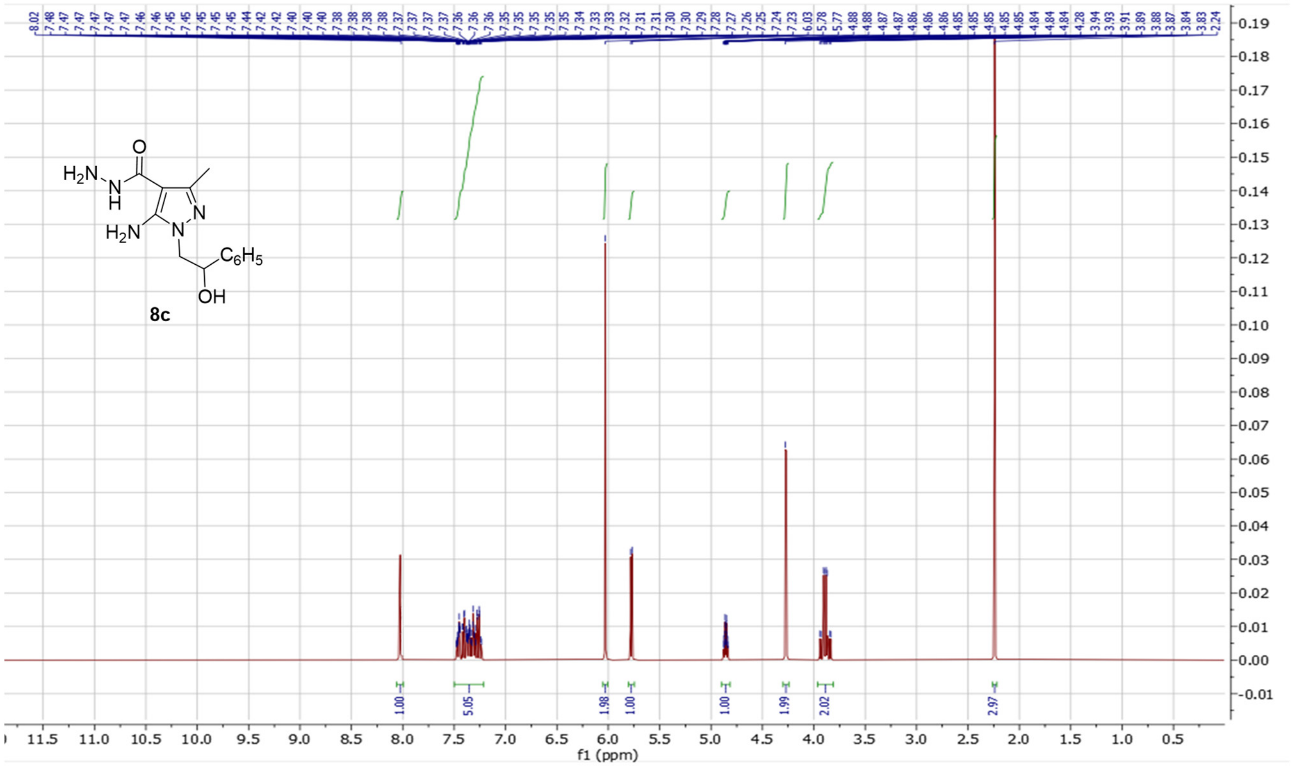

Figure S4: ^{13}C NMR (101 MHz) of compound **8c**

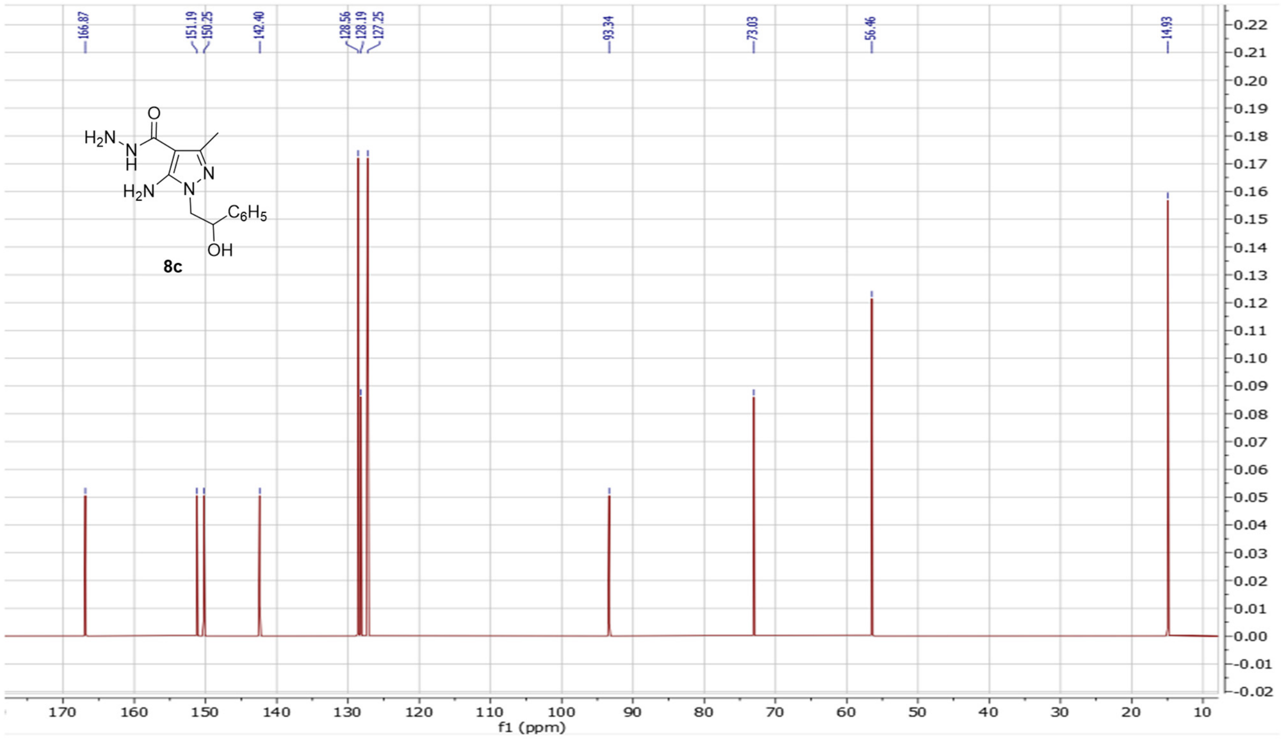

Figure S5:¹H NMR (400 MHz) of compound **8d**

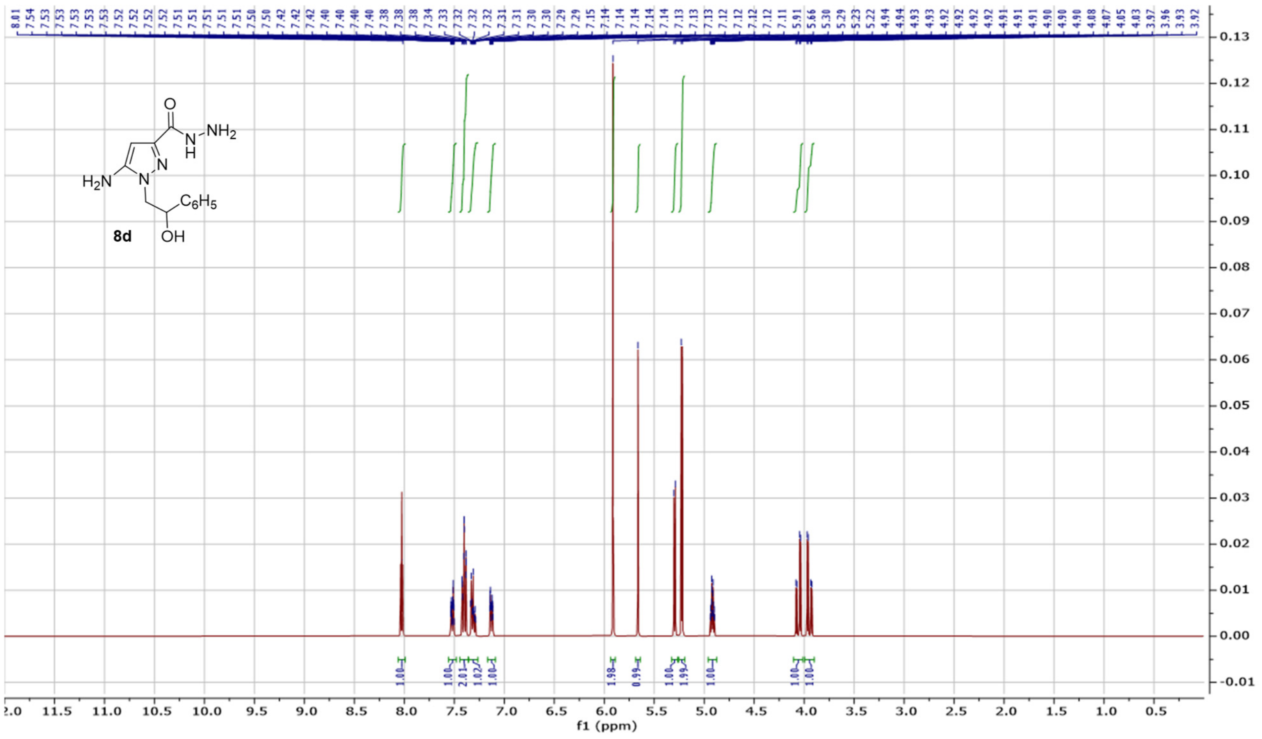

Figure S6: ¹³C NMR (101 MHz) of compound **8d**

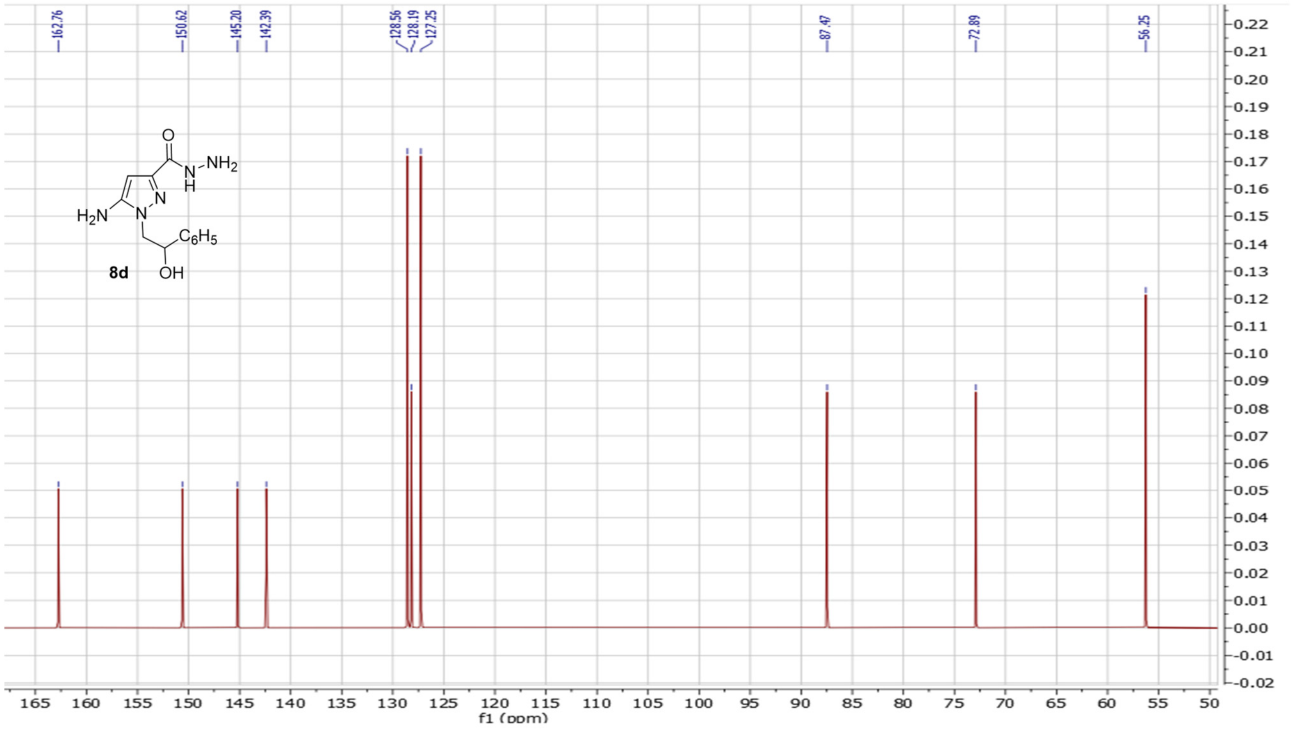

Figure S7: ¹H NMR (400 MHz) of compound **1a**

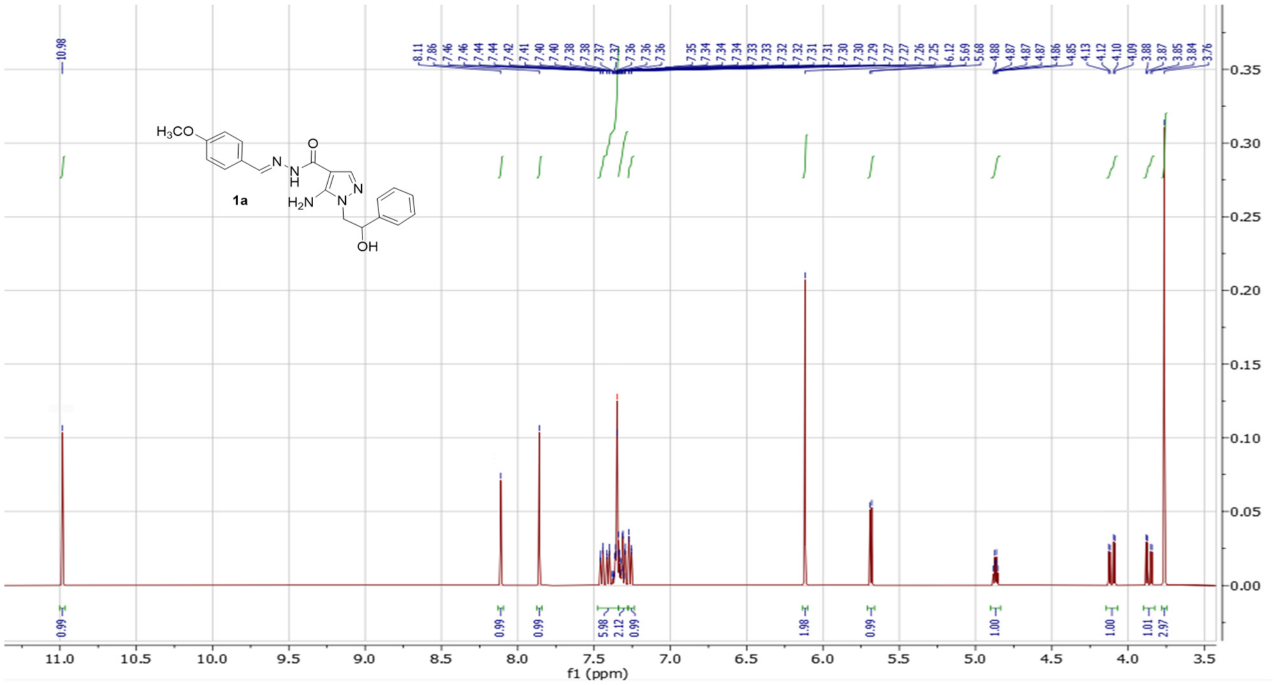

Figure S8: ¹³C NMR (101 MHz) of compound **1a**

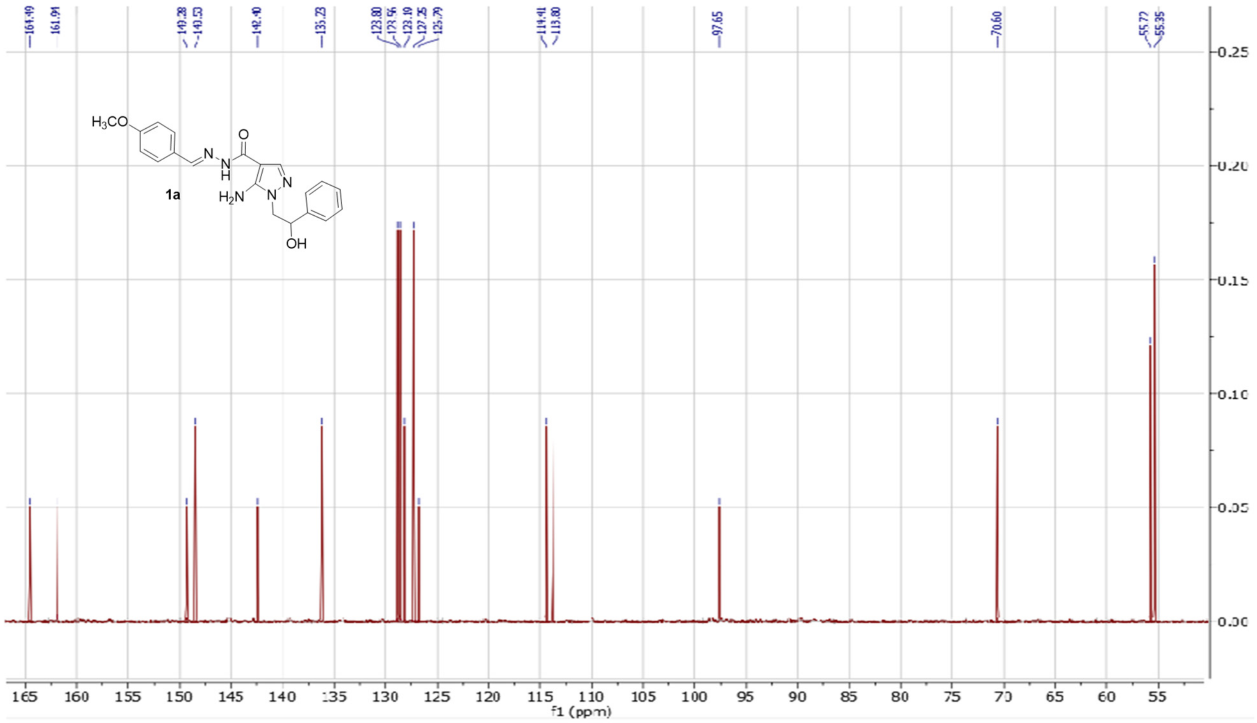

Figure S9:^{1}H NMR (400 MHz) of compound **1b**

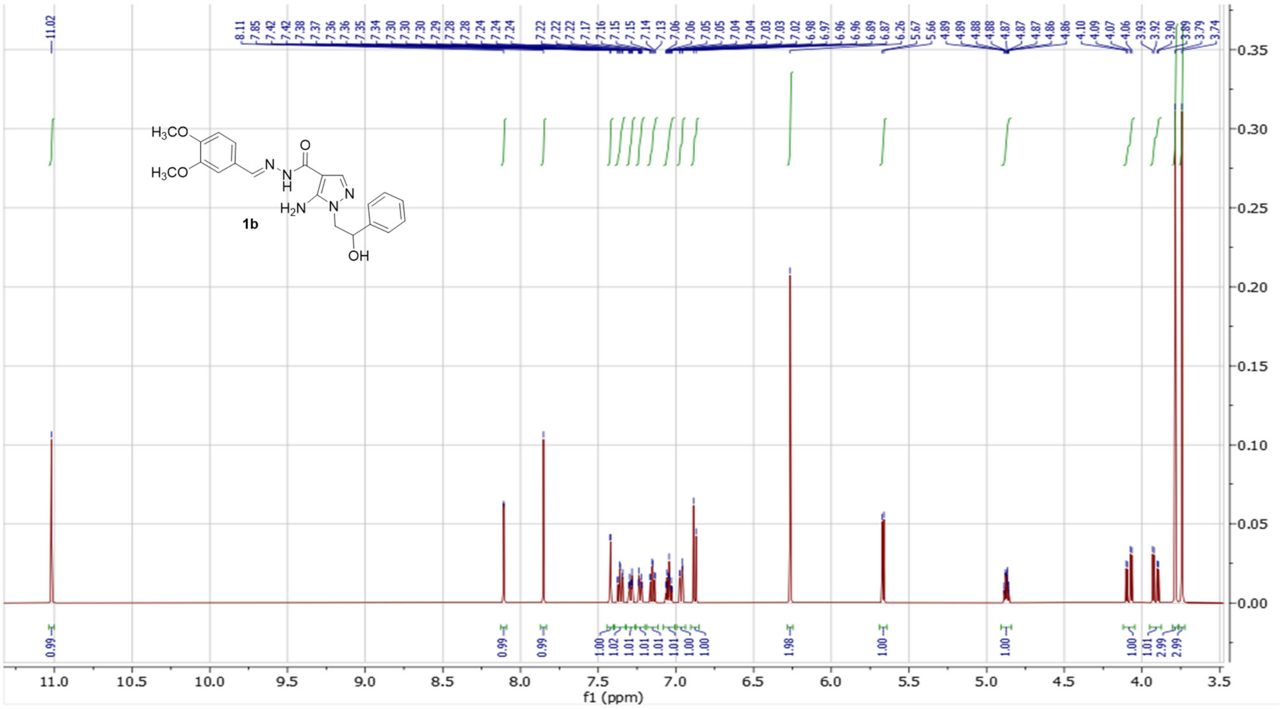

Figure S10: ^{13}C NMR (101 MHz) of compound **1b**

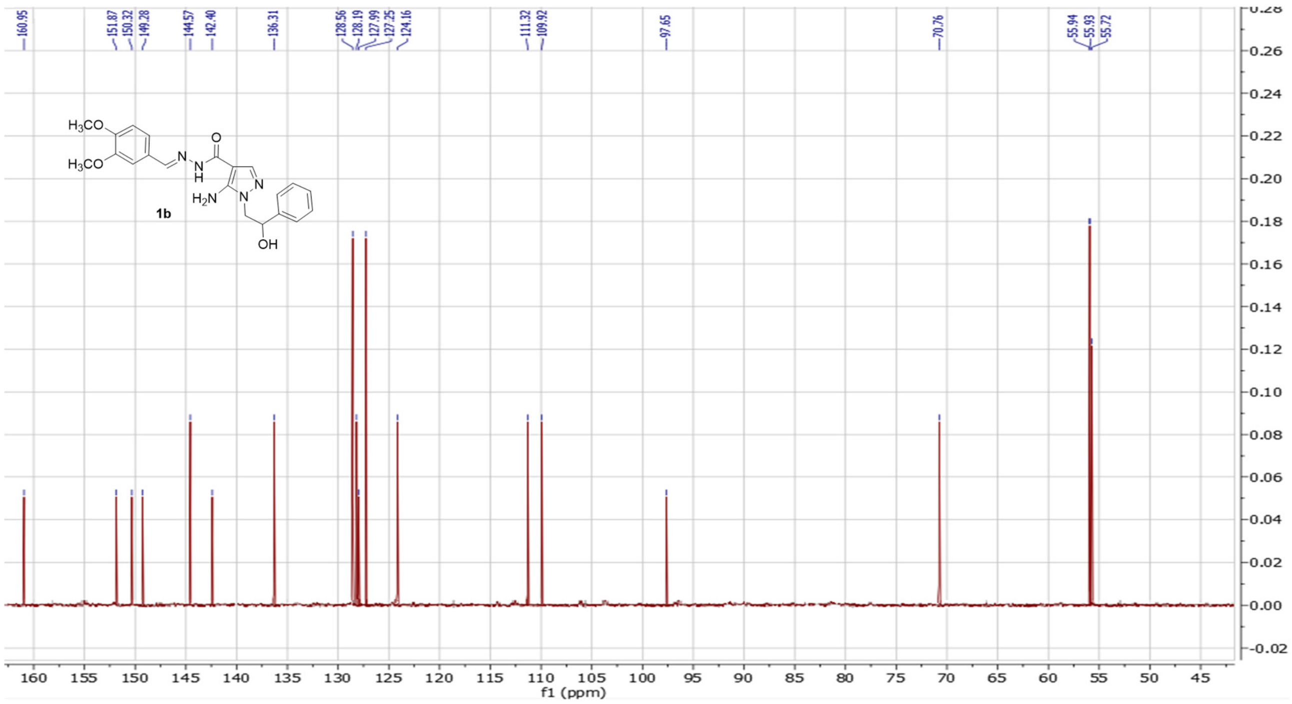

Figure S11:^{1}H NMR (400 MHz) of compound **1c**

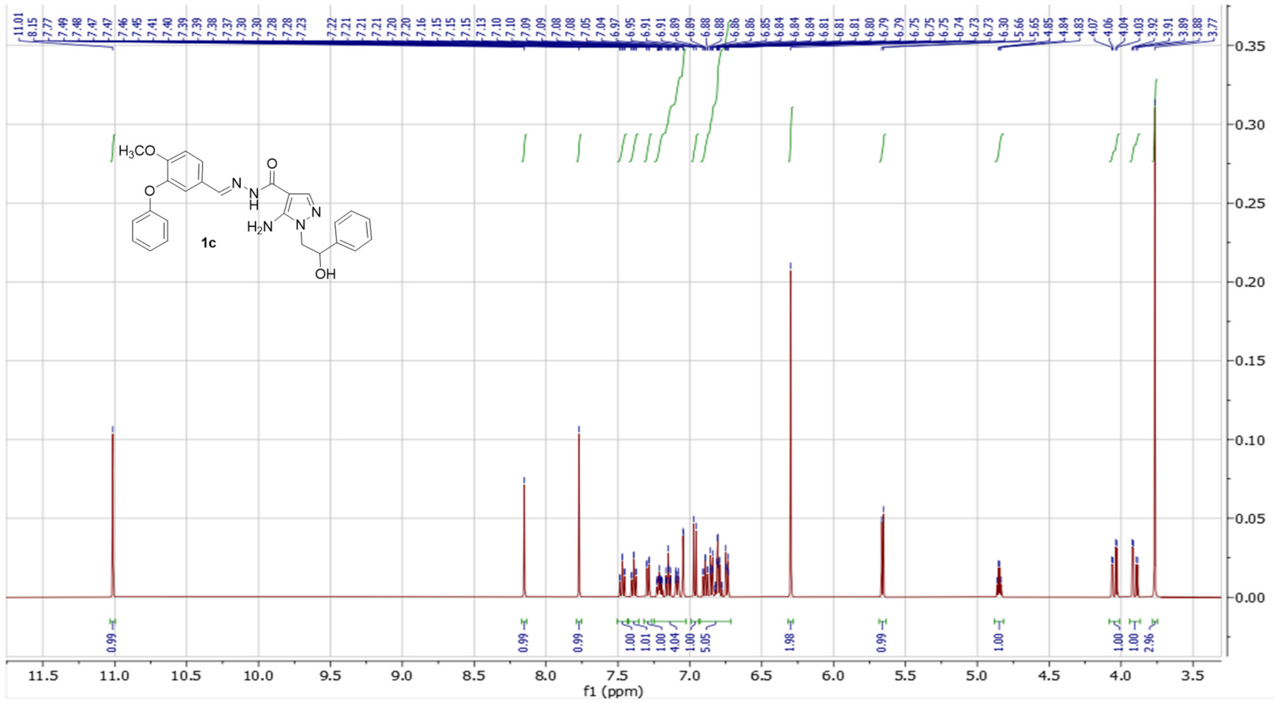

Figure S12: ^{13}C NMR (101 MHz) of compound **1c**

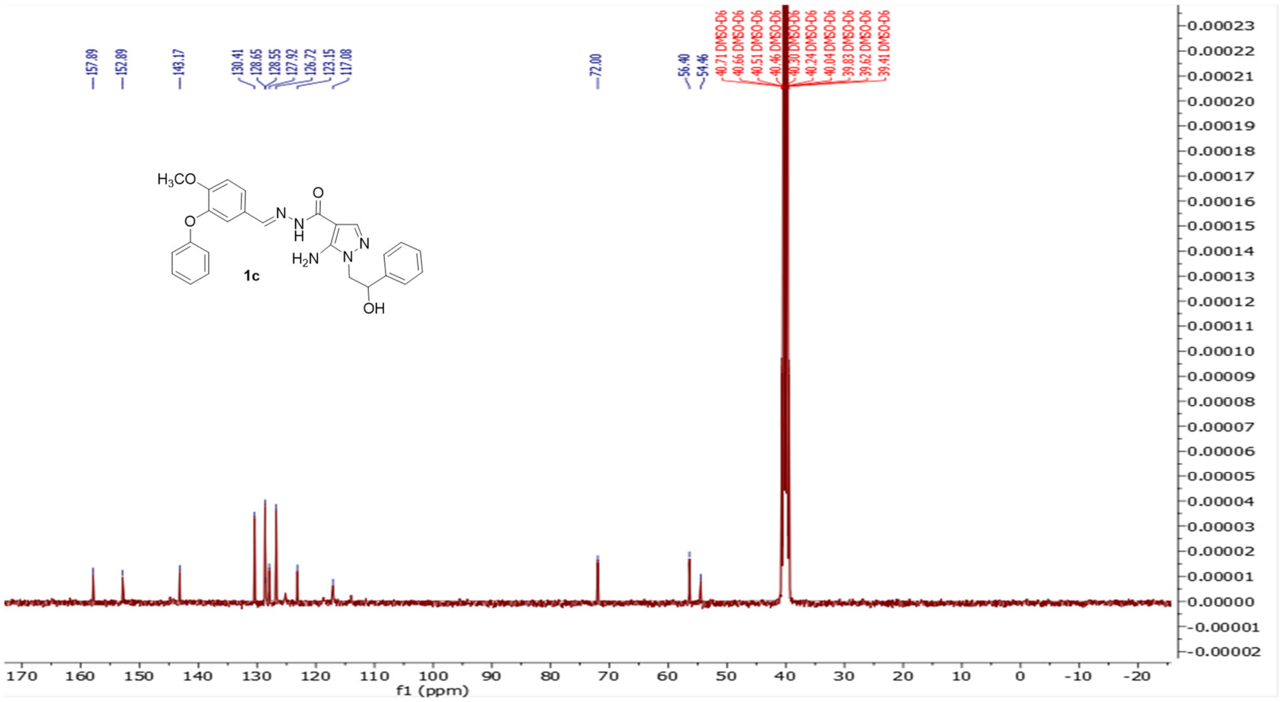

Figure S13:¹H NMR (400 MHz) of compound **1d**

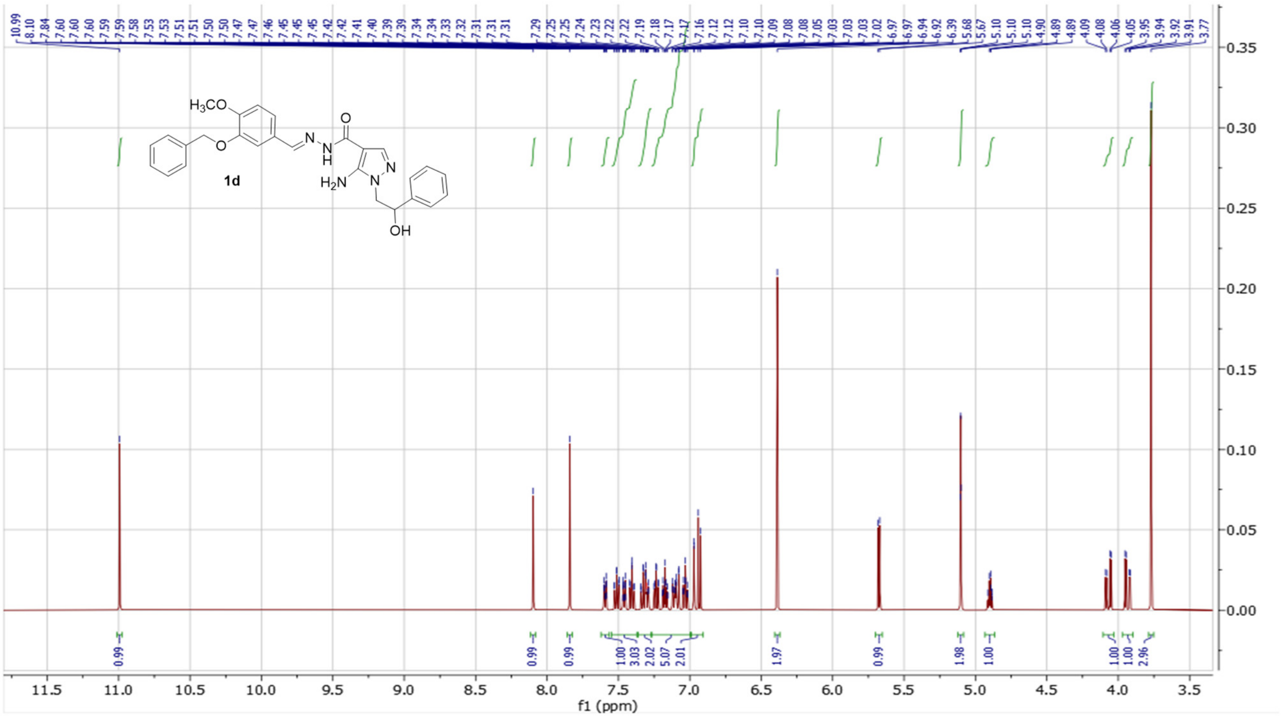

Figure S14: ¹³C NMR (101 MHz) of compound **1d**

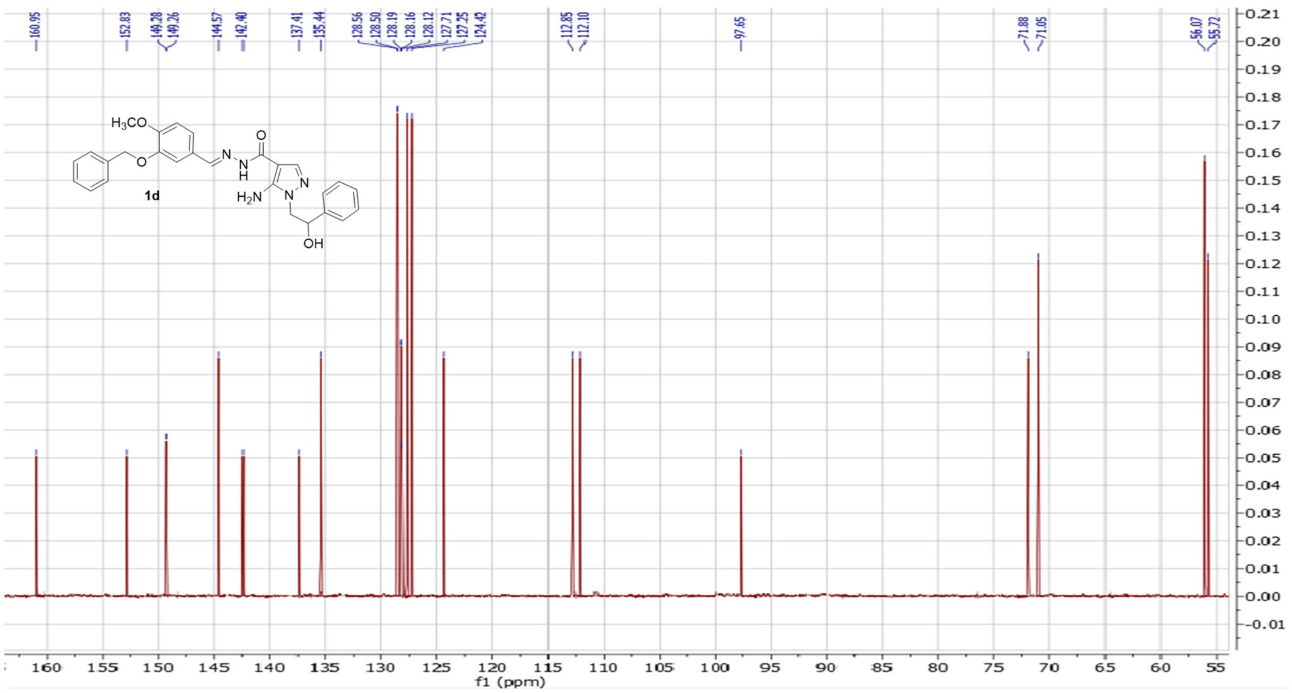

Figure S15: ^{1}H NMR (400 MHz) of compound **1e**

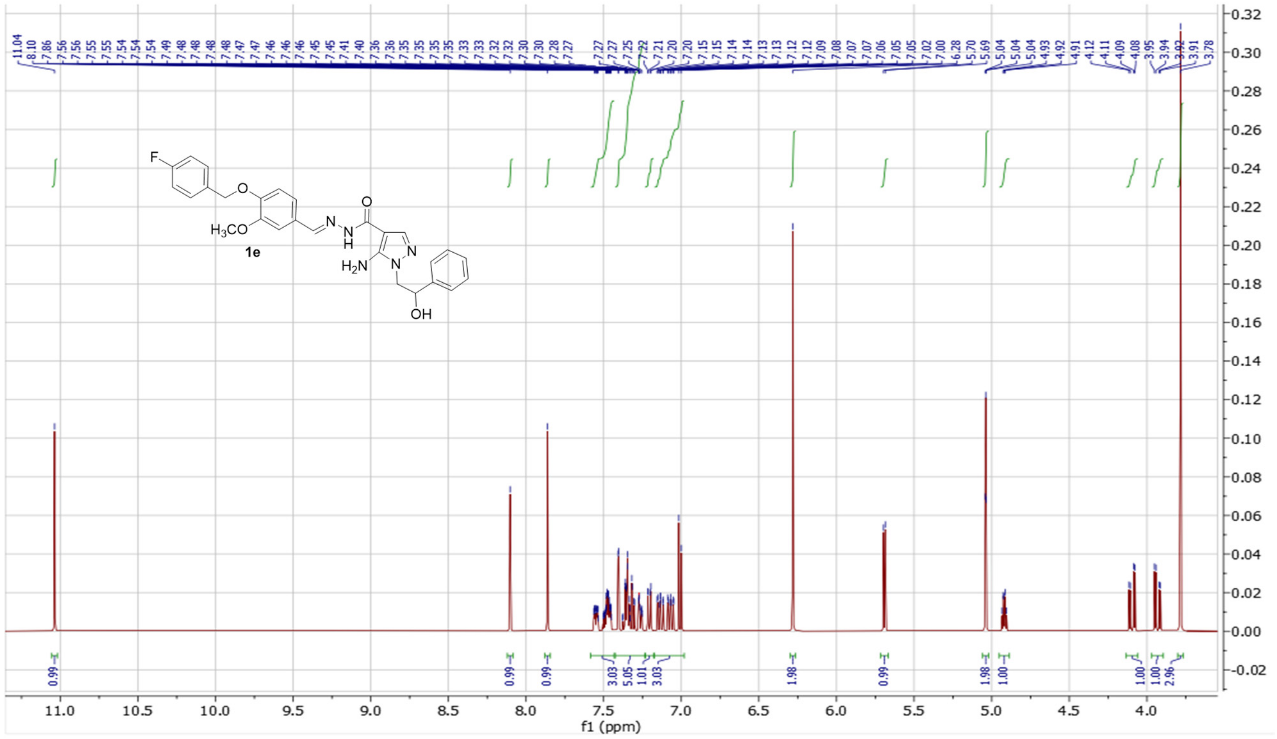

Figure S16: ^{13}C NMR (101 MHz) of compound **1e**

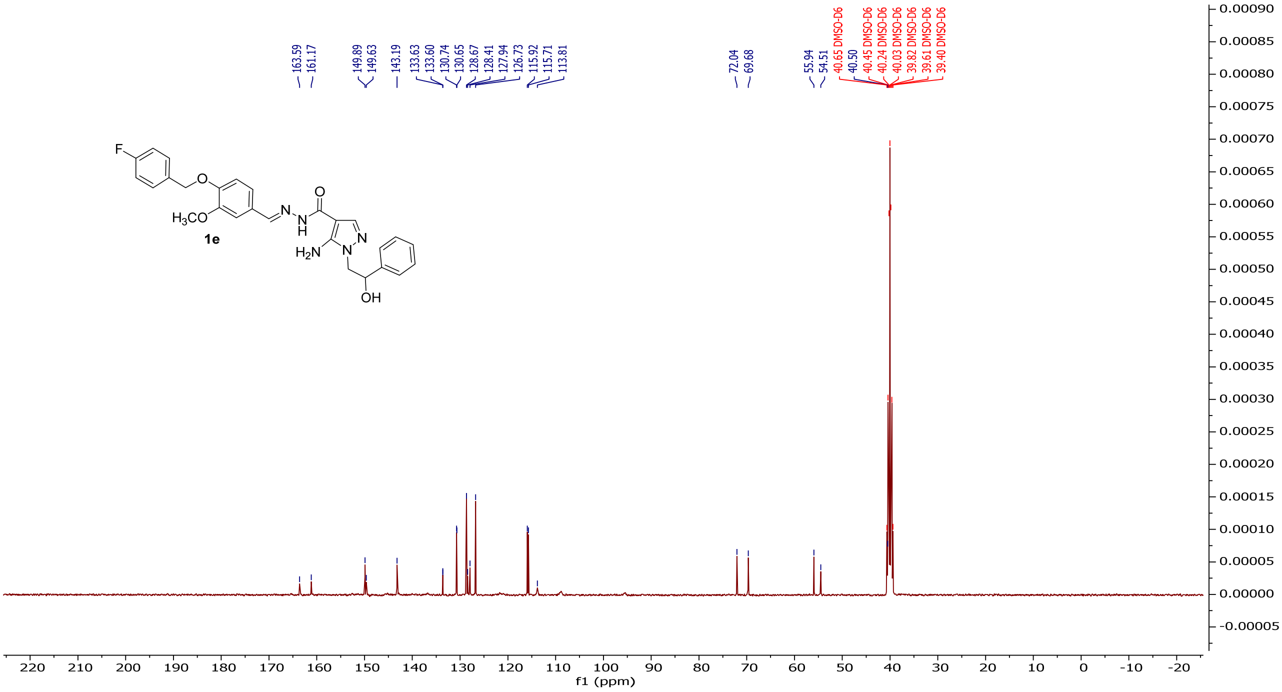

Figure S17:^{1}H NMR (400 MHz) of compound **1f**

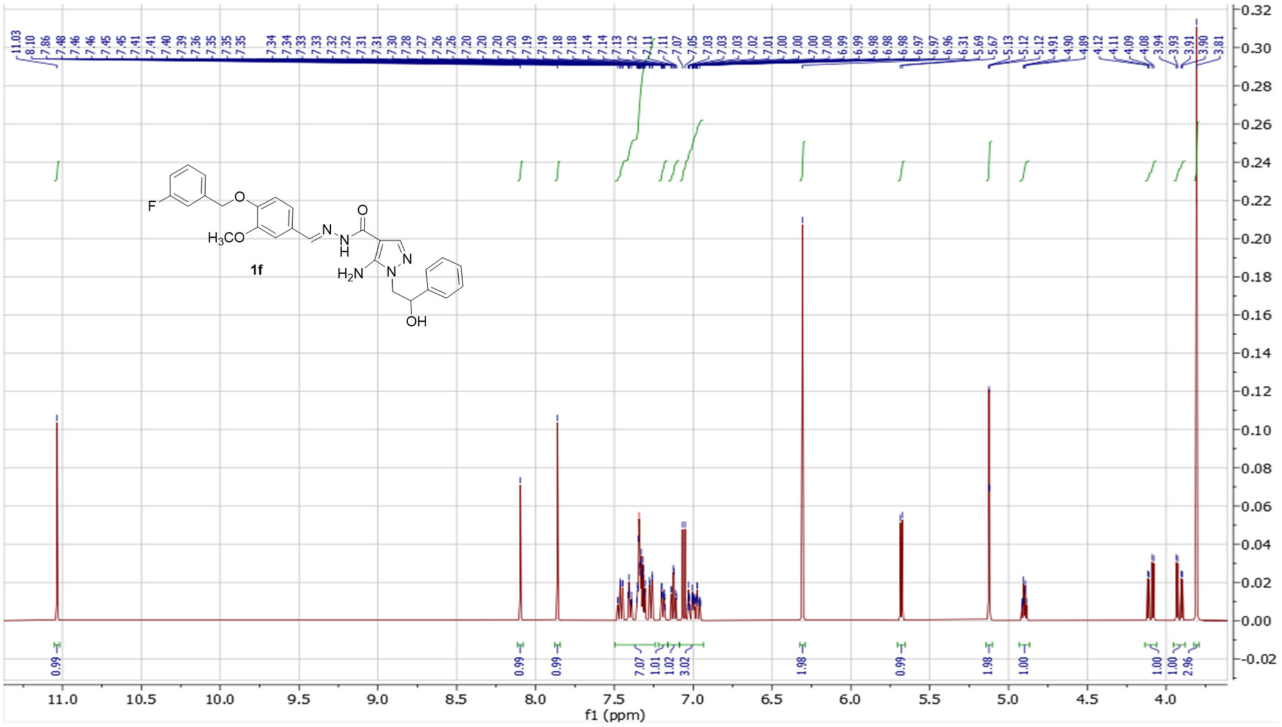

Figure S18: ^{13}C NMR (101 MHz) of compound **1f**

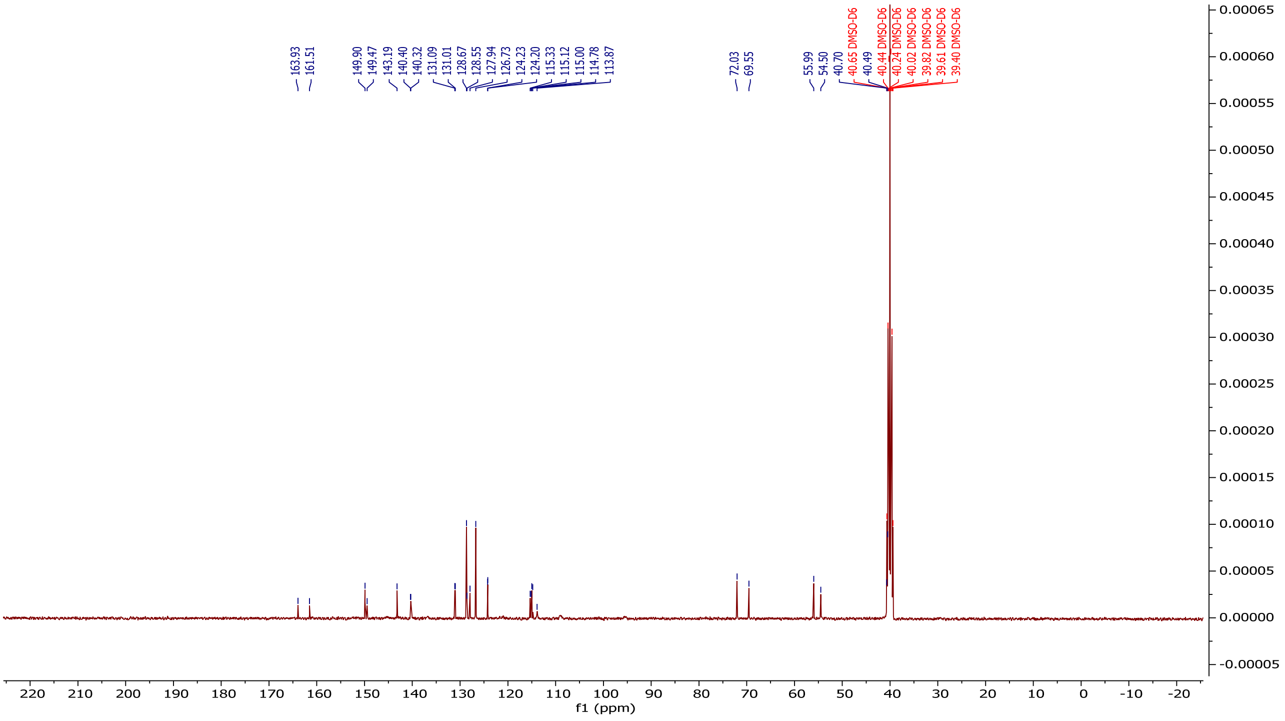

Figure S19:^{1}H NMR (400 MHz) of compound **1g**

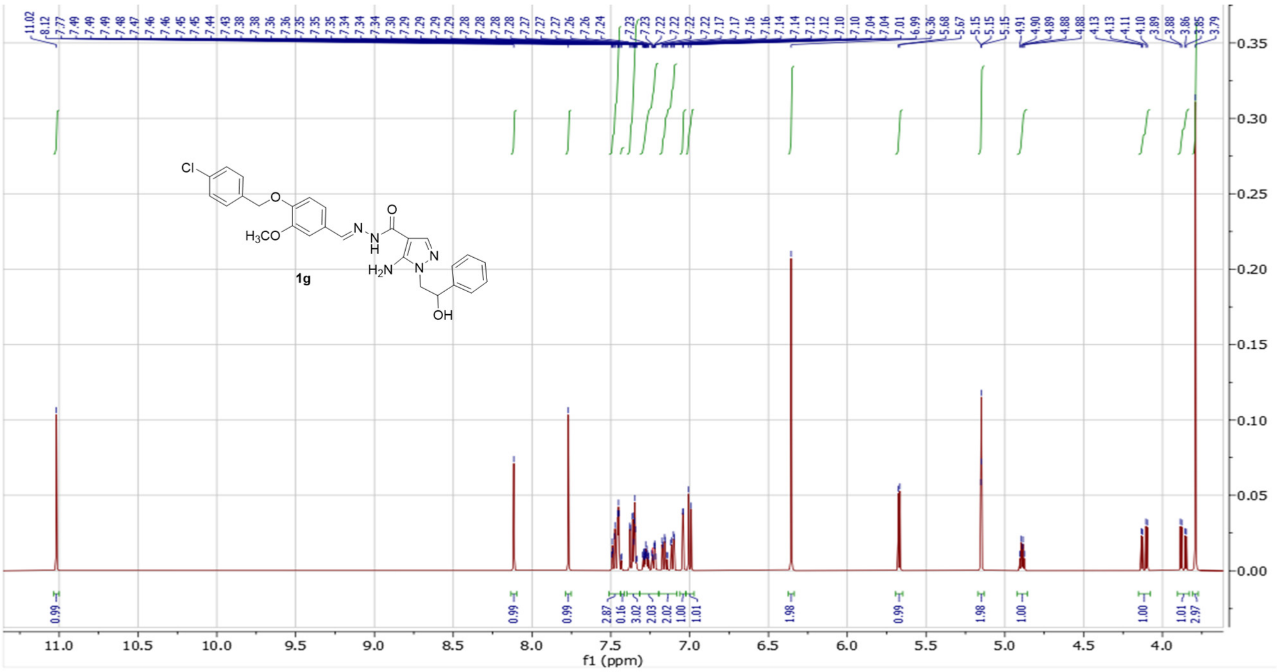

Figure S20: ^{13}C NMR (101 MHz) of compound **1g**

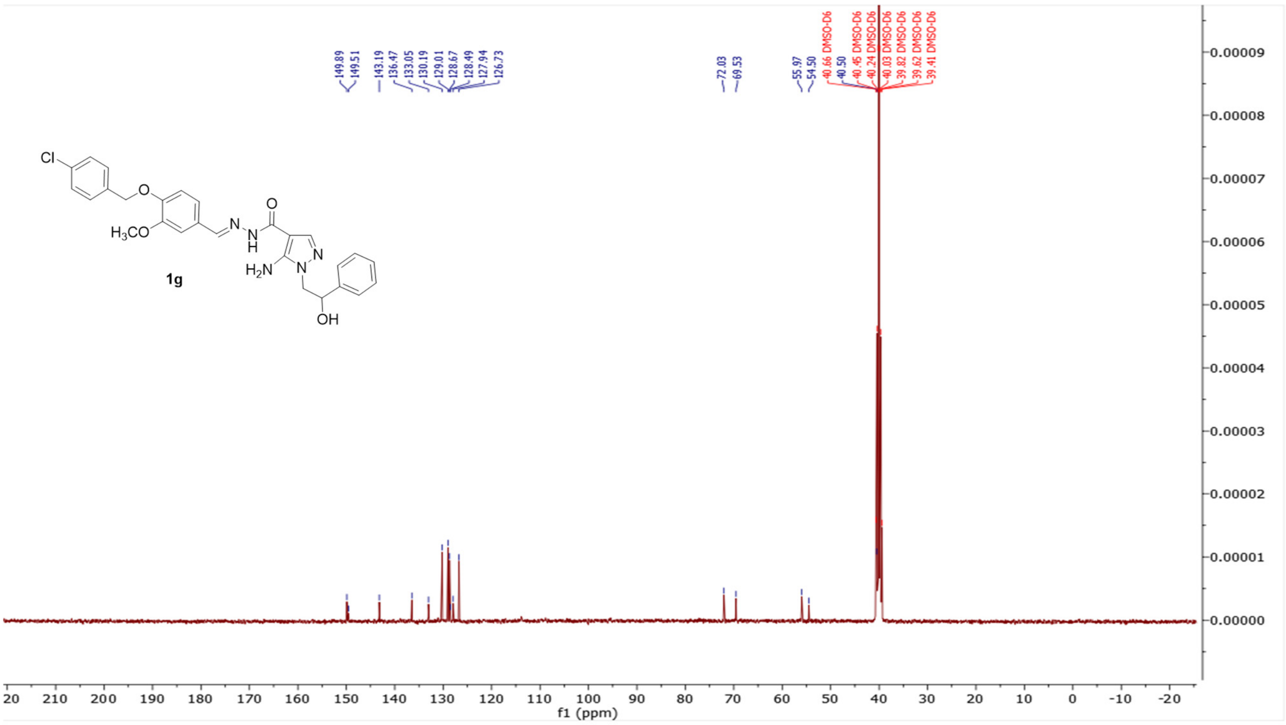

Figure S21:^{1}H NMR (400 MHz) of compound **1h**

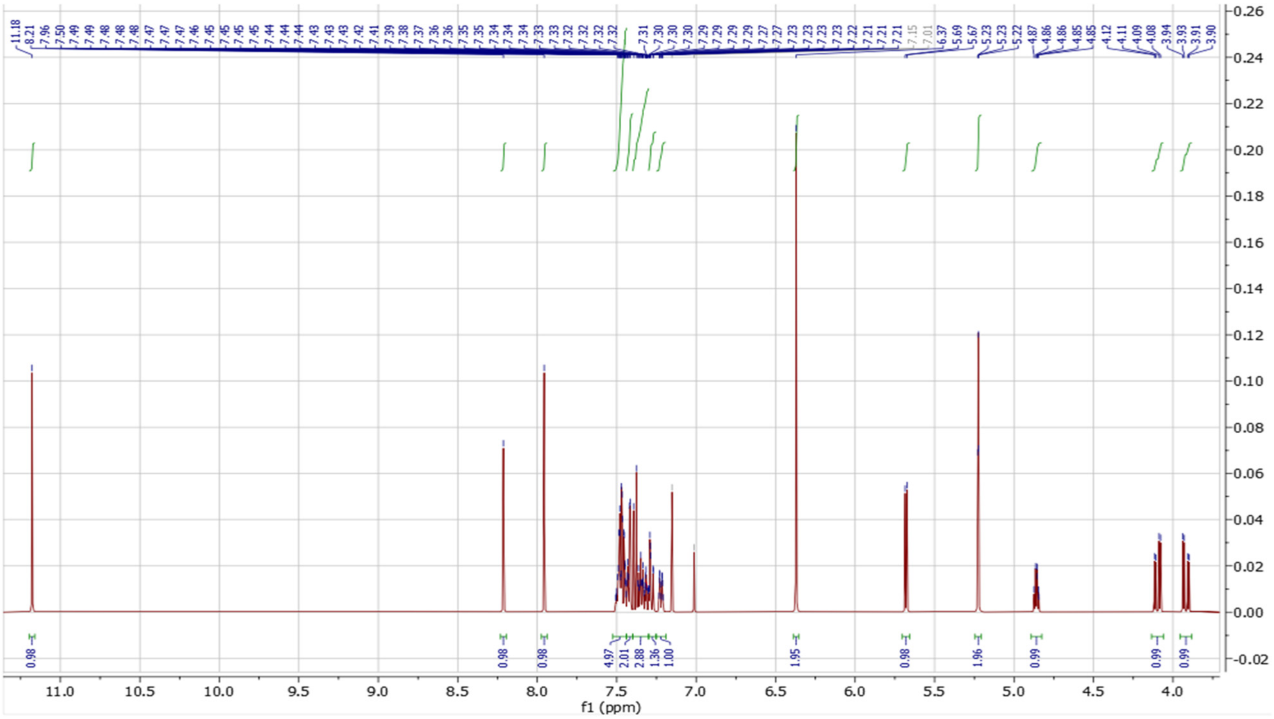

Figure S22: ^{13}C NMR (101 MHz) of compound **1h**

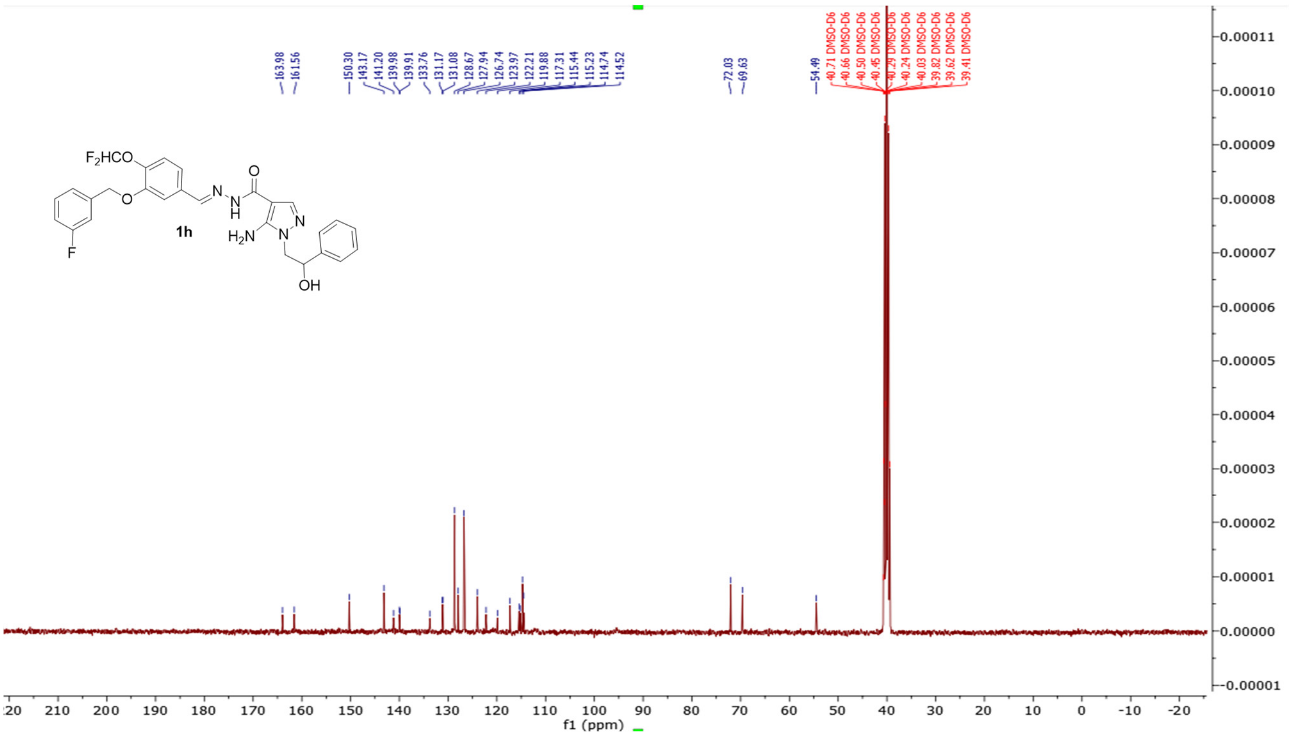

Figure S23: ^{1}H NMR (400 MHz) of compound **1i**

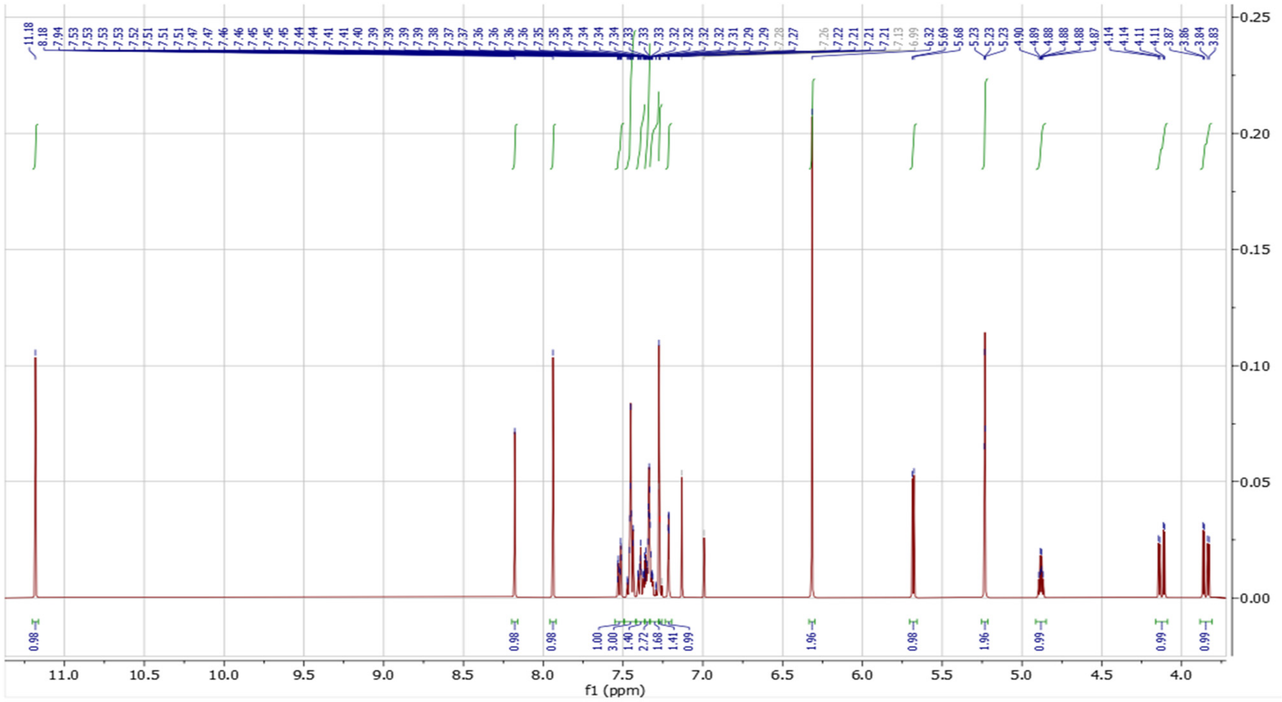

Figure S24: ^{13}C NMR (101 MHz) of compound **1i**

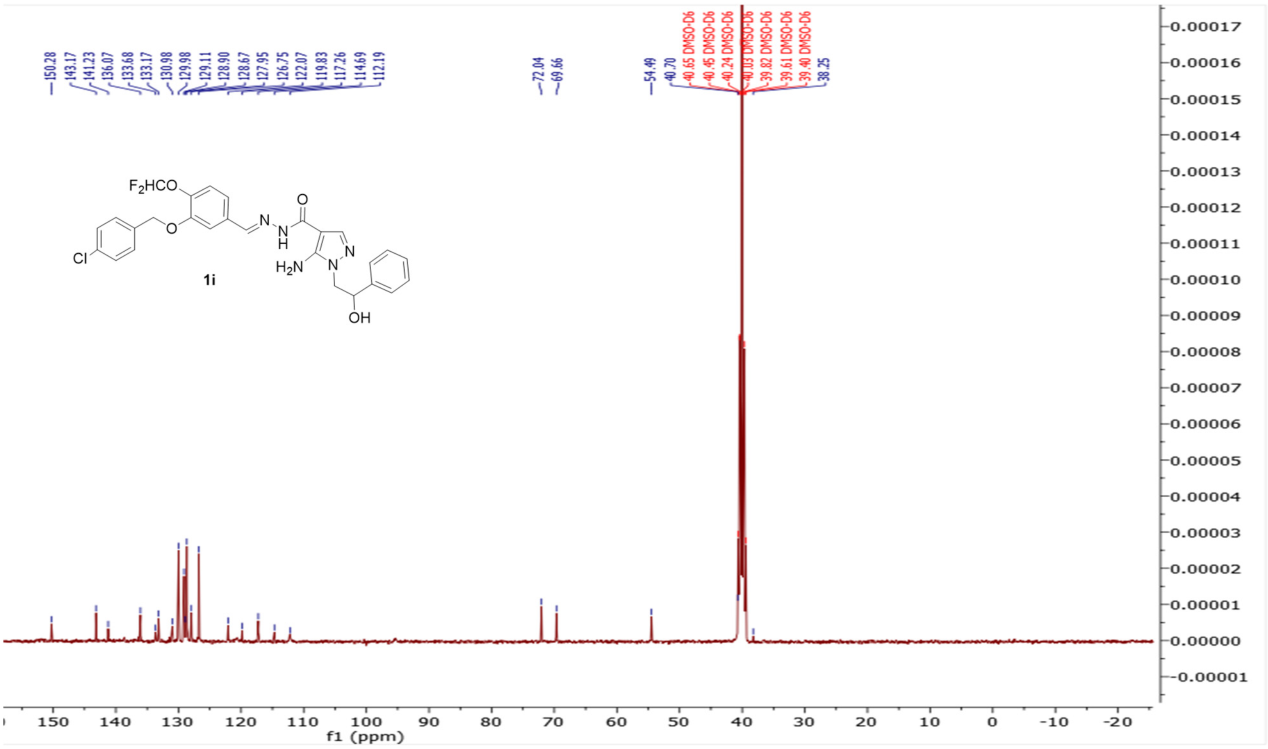

Figure S25:¹H NMR (400 MHz) of compound **2a**

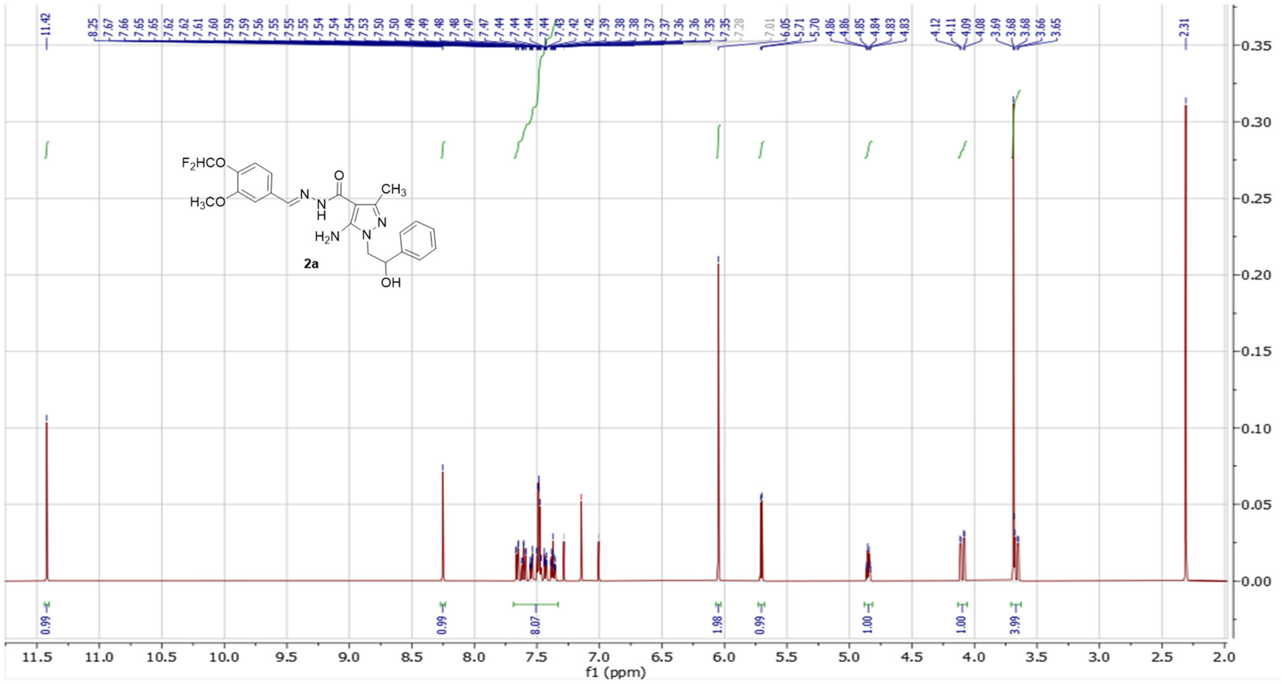

Figure S26: ¹³C NMR (101 MHz) of compound **2a**

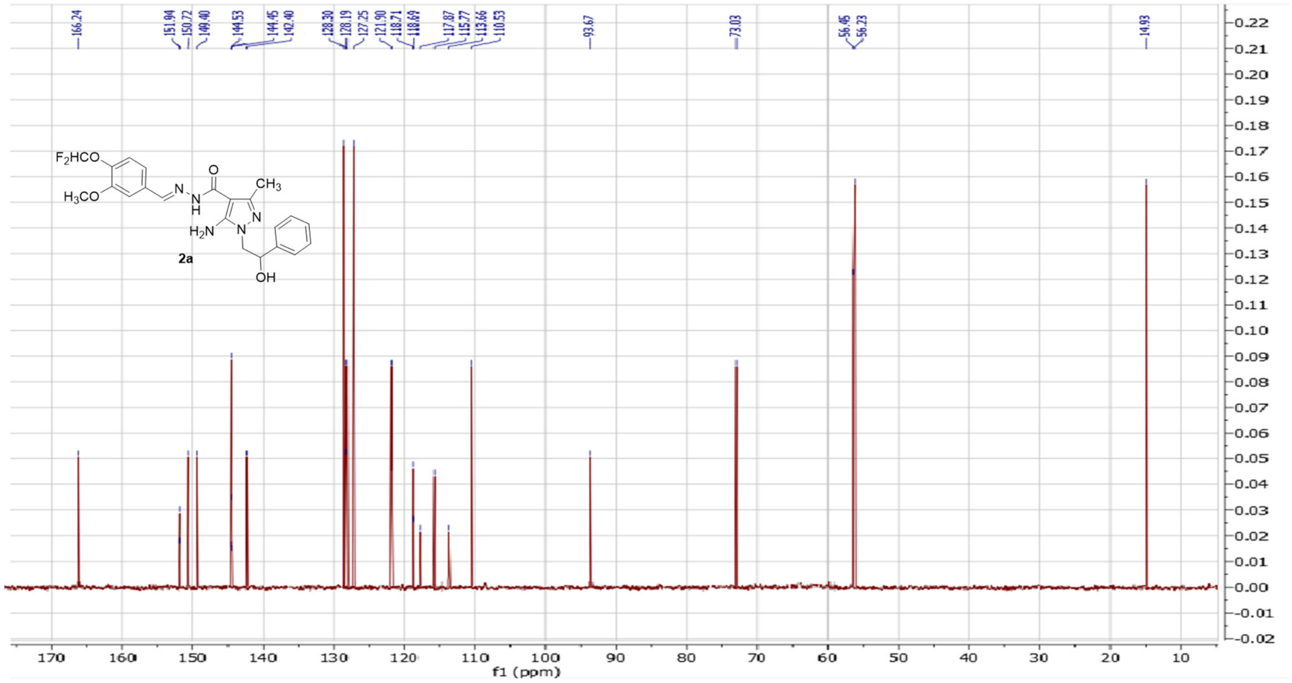

Figure S27: ¹H NMR (400 MHz) of compound **2b**

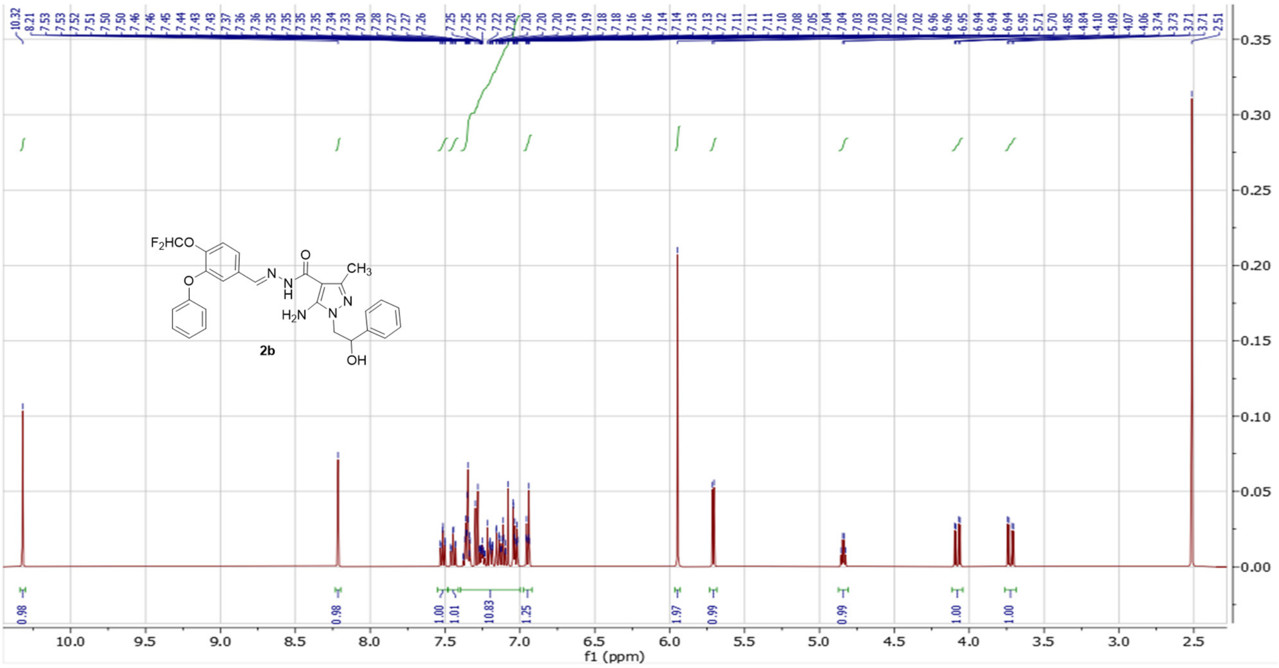

Figure S28: ¹³C NMR (101 MHz) of compound **2b**

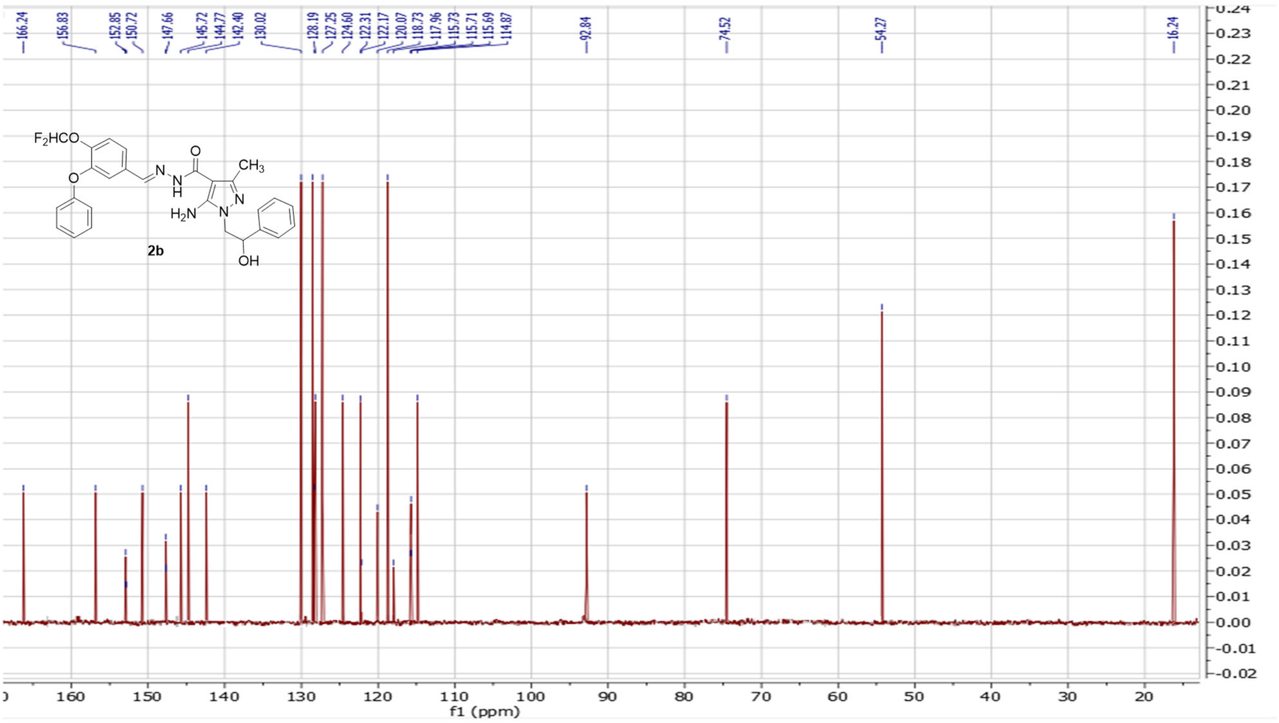

Figure S29:^{1}H NMR (400 MHz) of compound **2c**

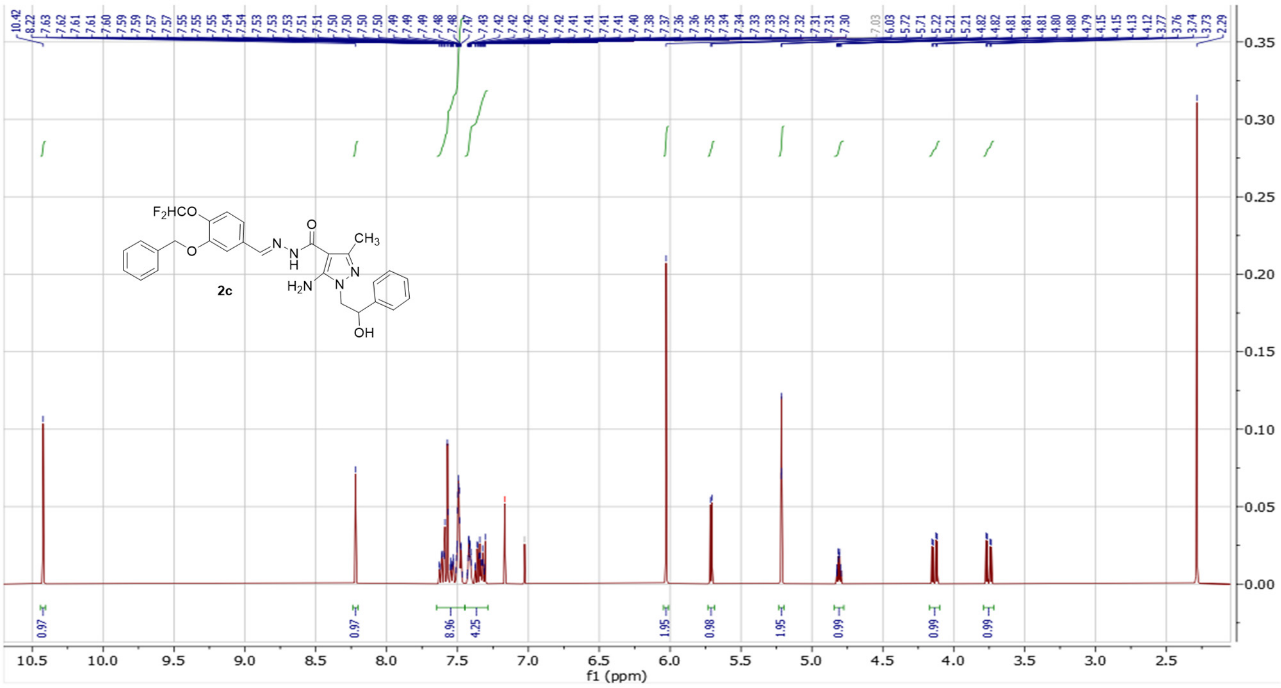

Figure S30: ^{13}C NMR (100 MHz) of compound **2c**

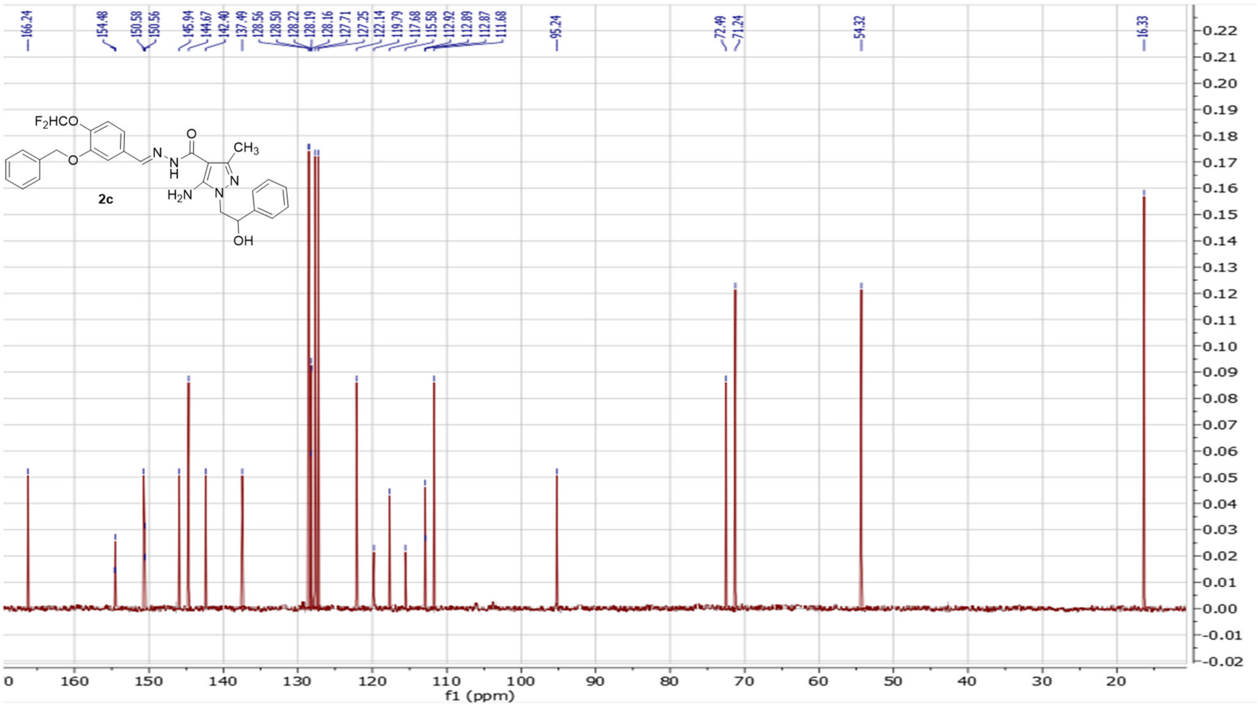

Figure S31:¹H NMR (400 MHz) of compound **3a**

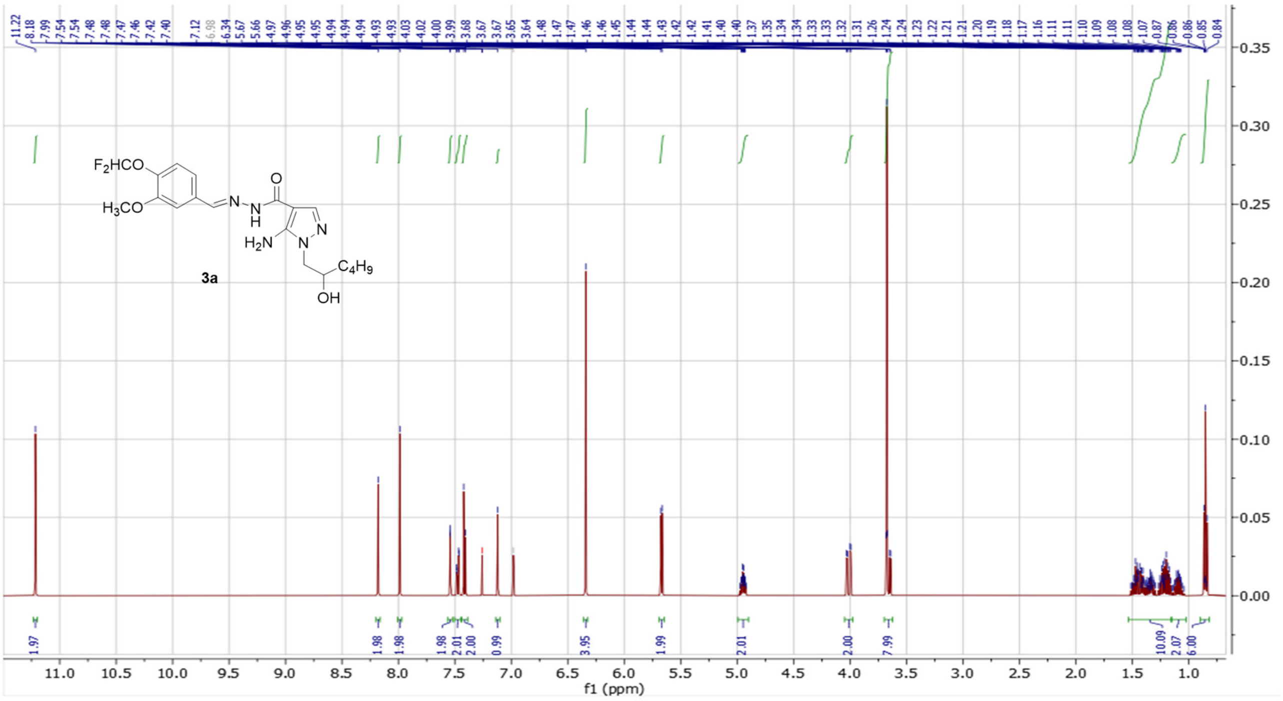

Figure S32: ¹³C NMR (101 MHz) of compound **3a**

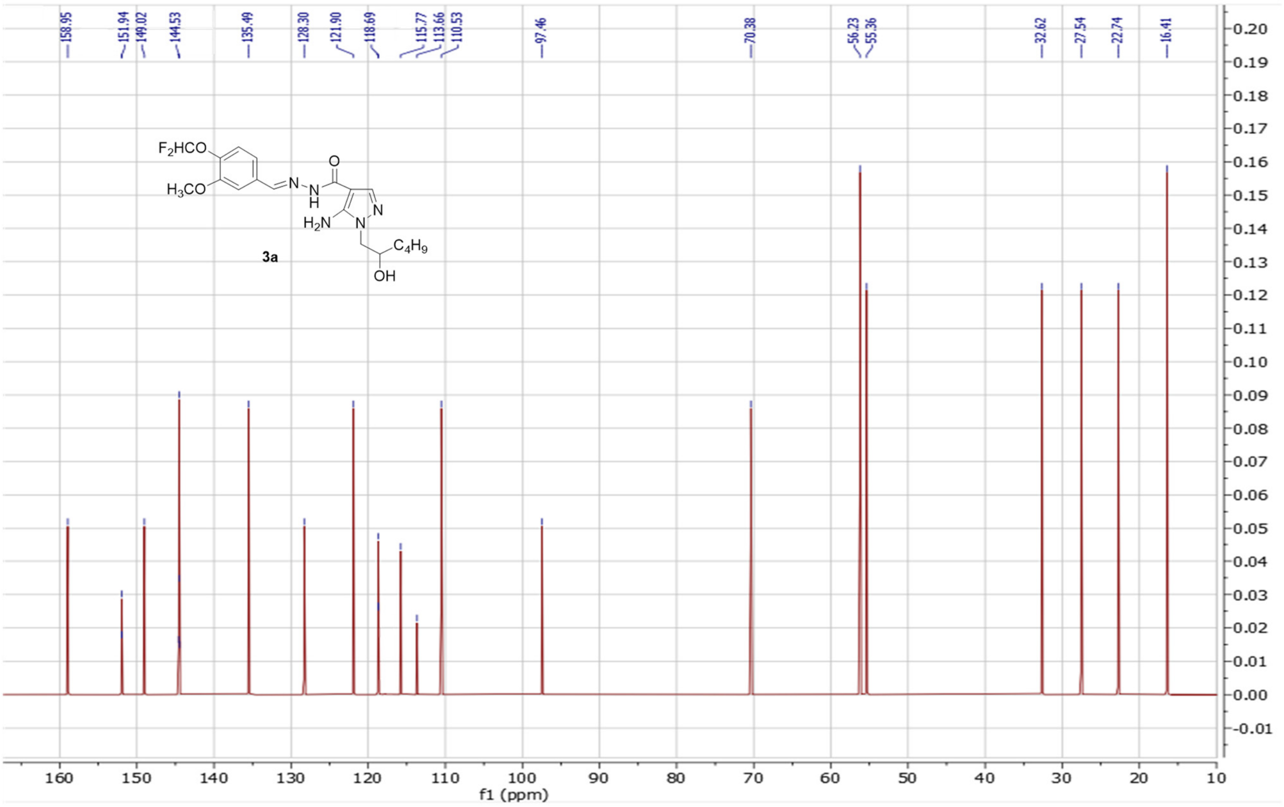

Figure S33:^{1}H NMR (400 MHz) of compound **3b**

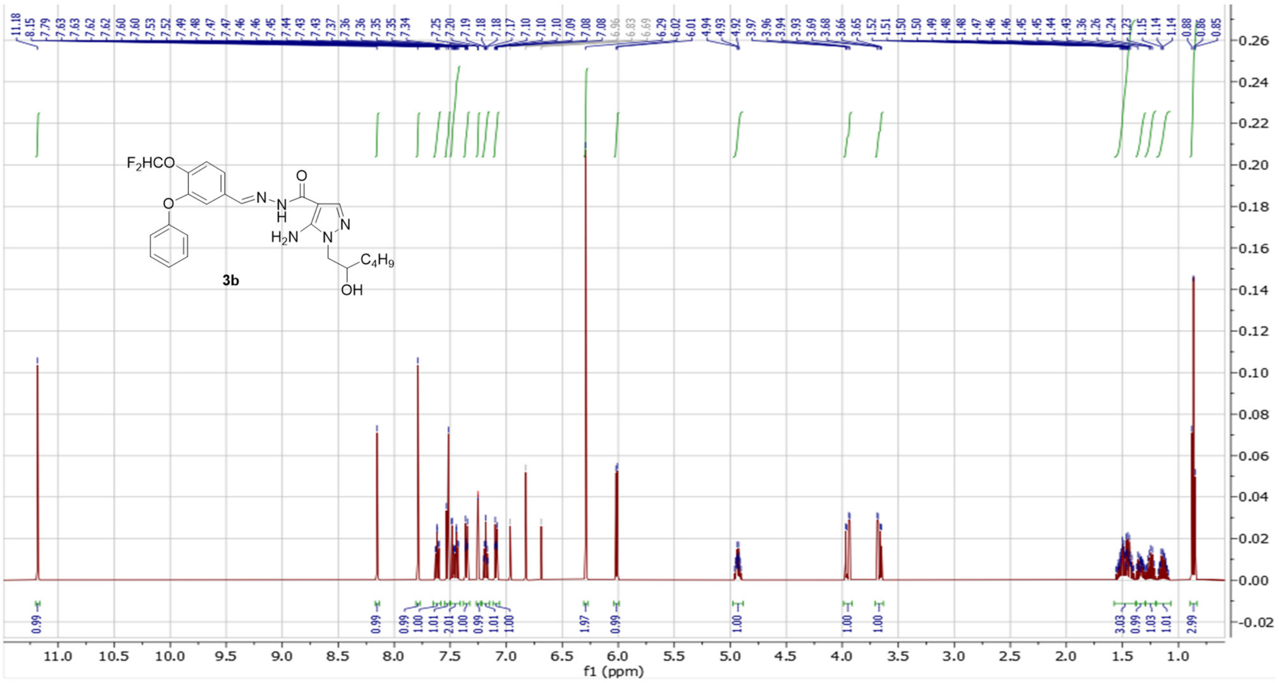

Figure S34: ^{13}C NMR (101 MHz) of compound **3b**

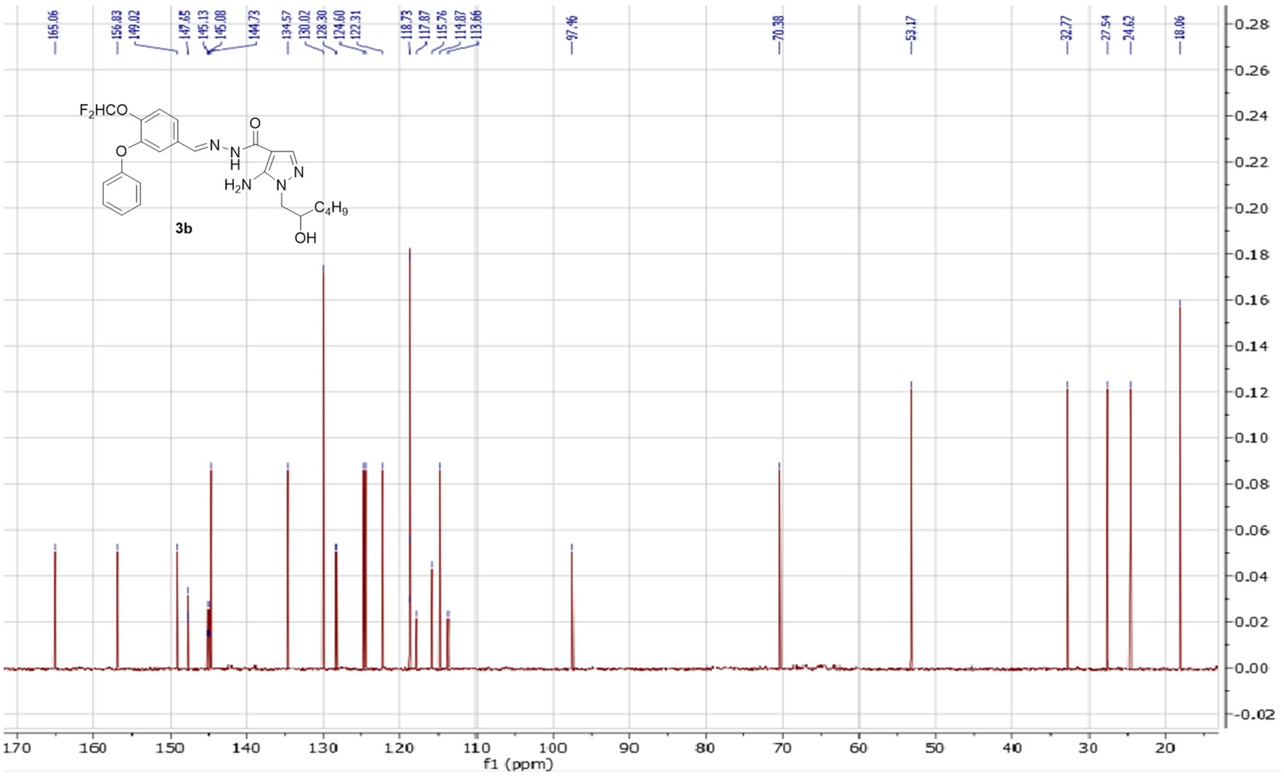

Figure S35:¹H NMR (400 MHz) of compound **3c**

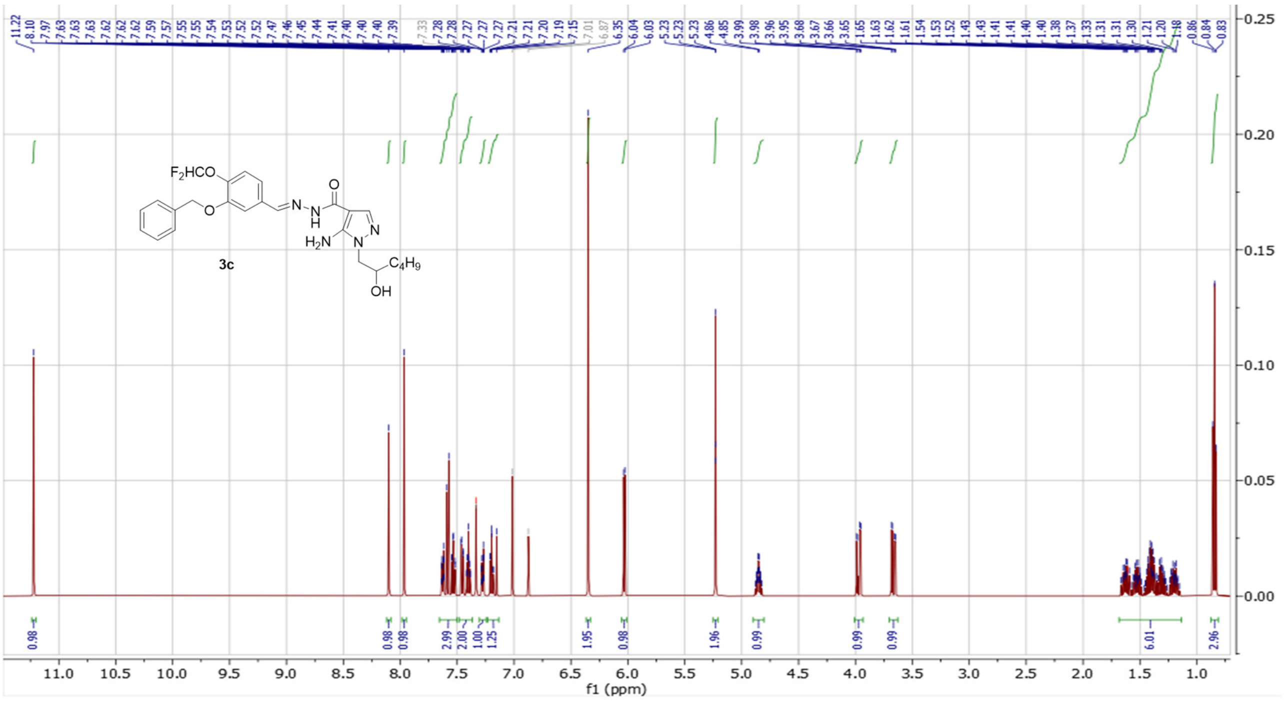

Figure S36: ¹³C NMR (101 MHz) of compound **3c**

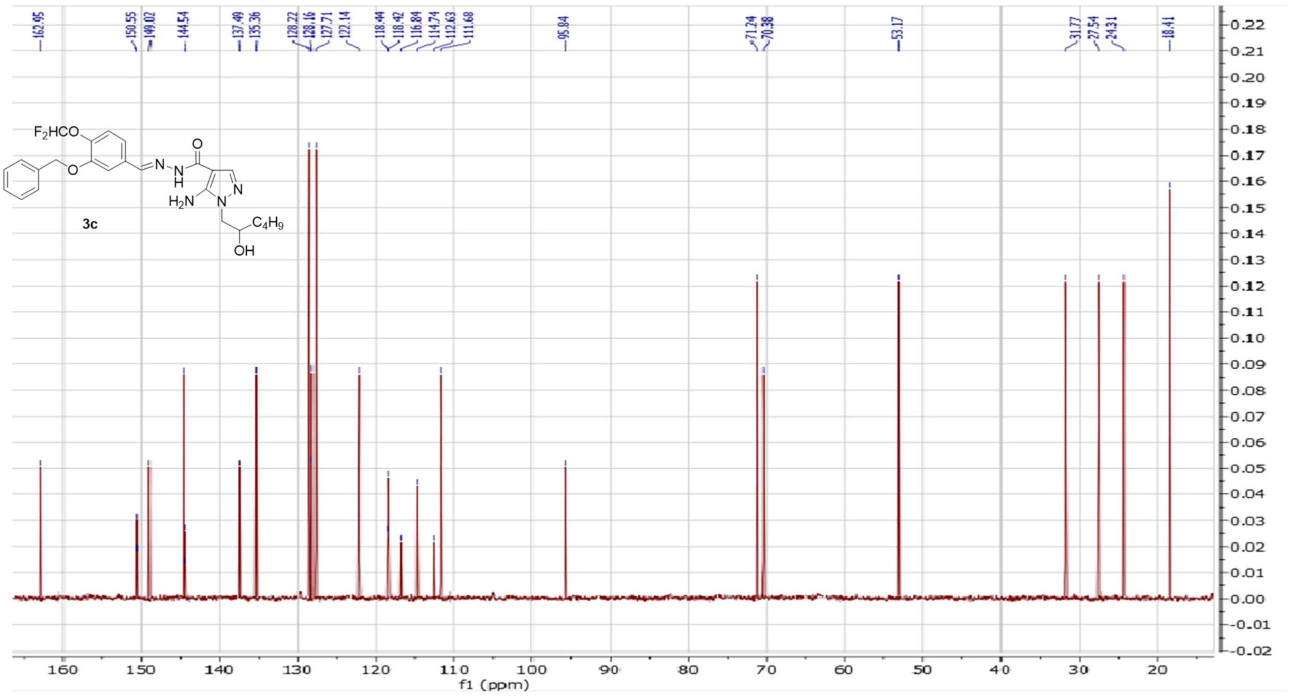

Figure S37:^{1}H NMR (400 MHz) of compound **4a**

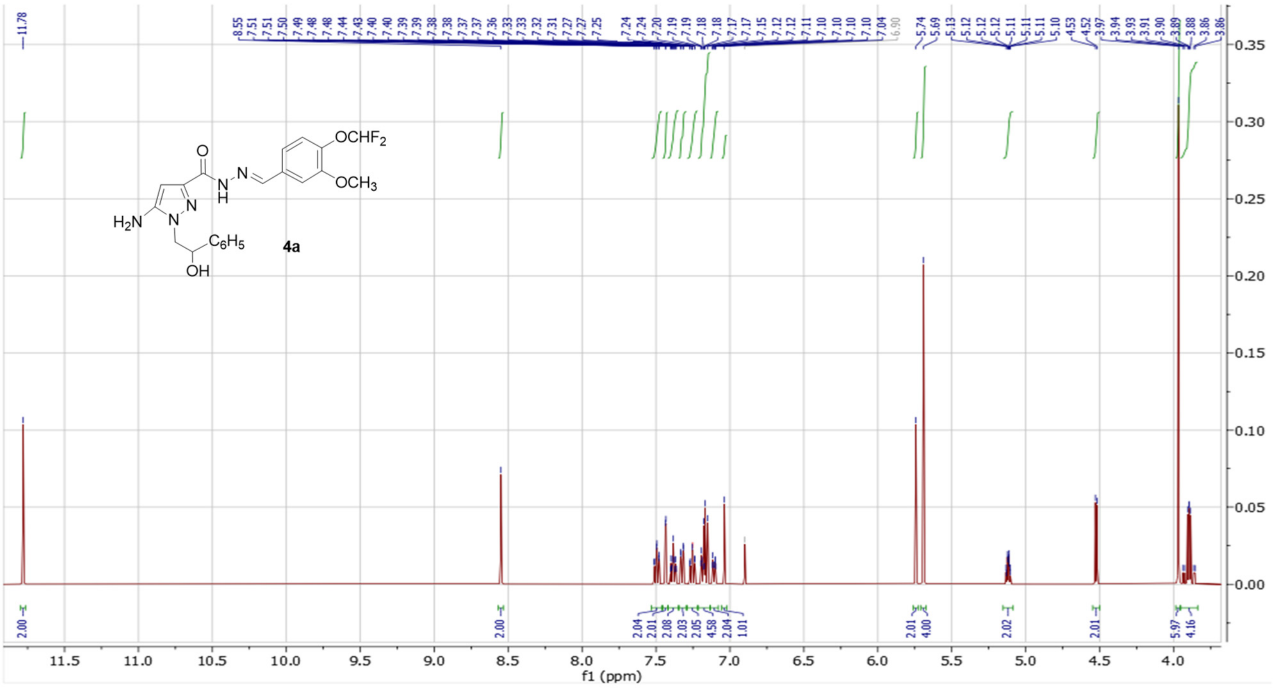

Figure S38: ^{13}C NMR (101 MHz) of compound **4a**

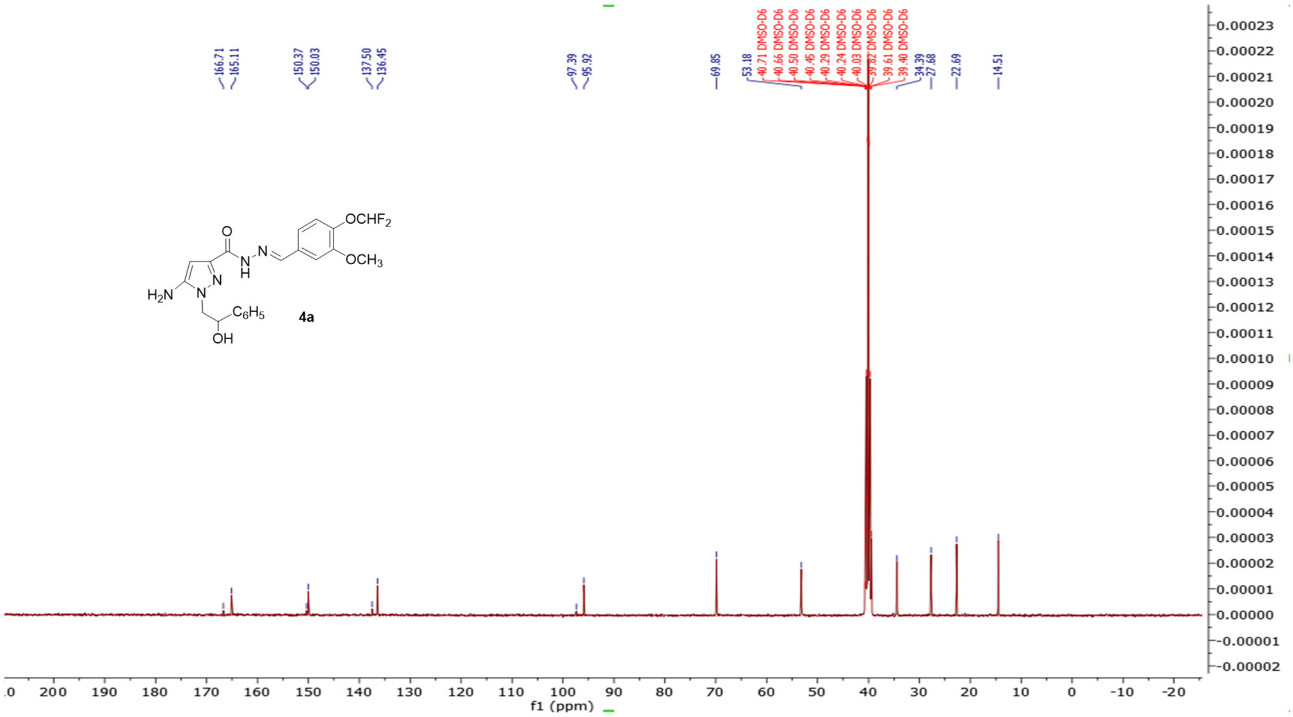

Figure S39:^{1}H NMR (400 MHz) of compound **4b**

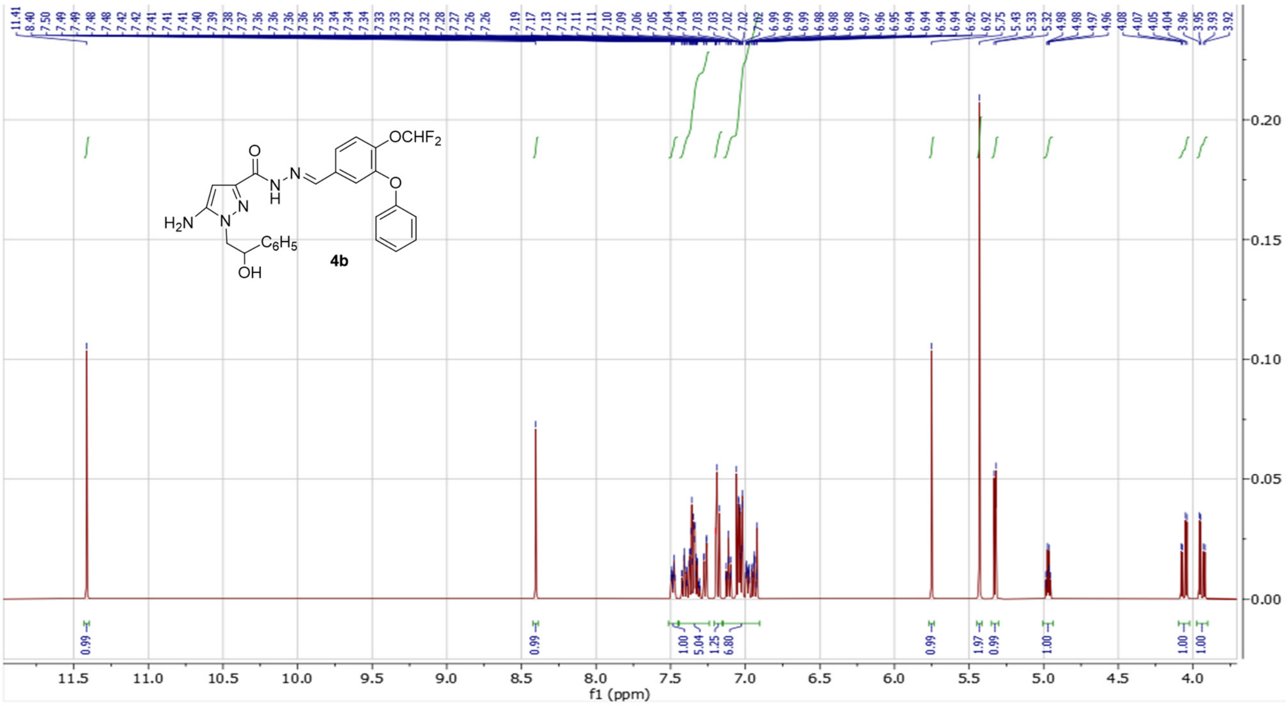

Figure S40: ^{13}C NMR (101 MHz) of compound **4b**

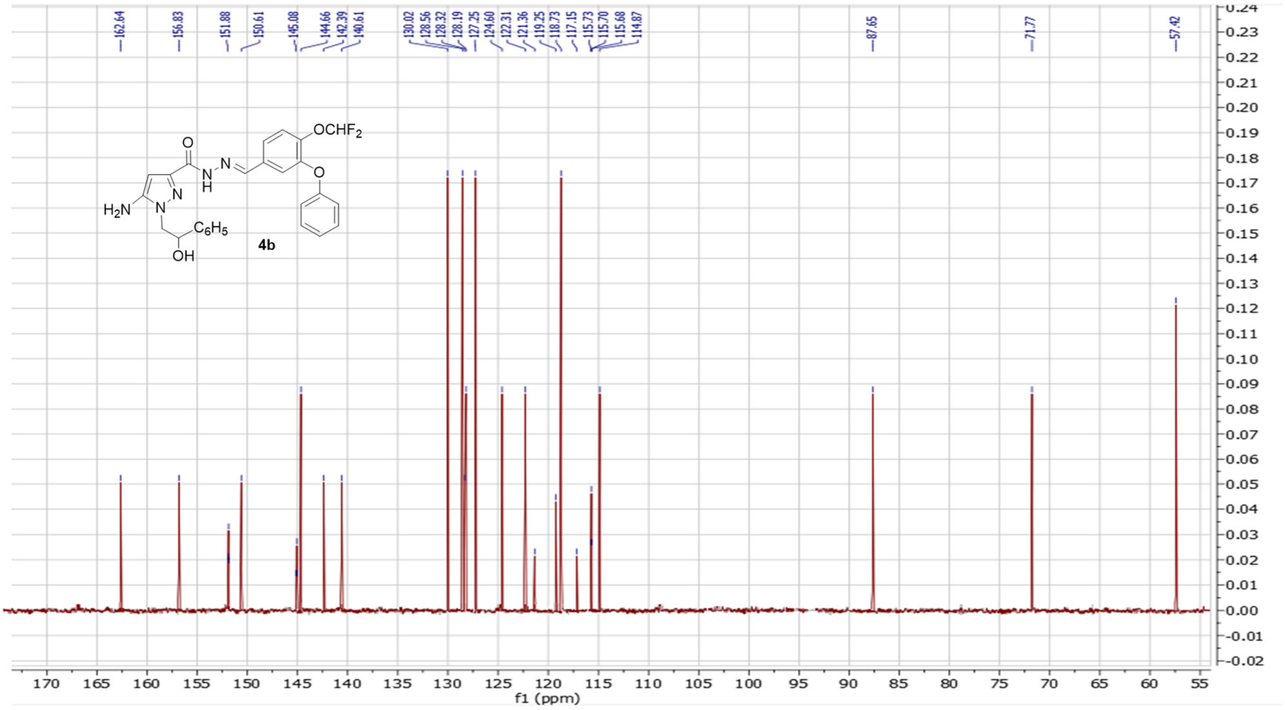

Figure S41:^{1}H NMR (400 MHz) of compound **4c**

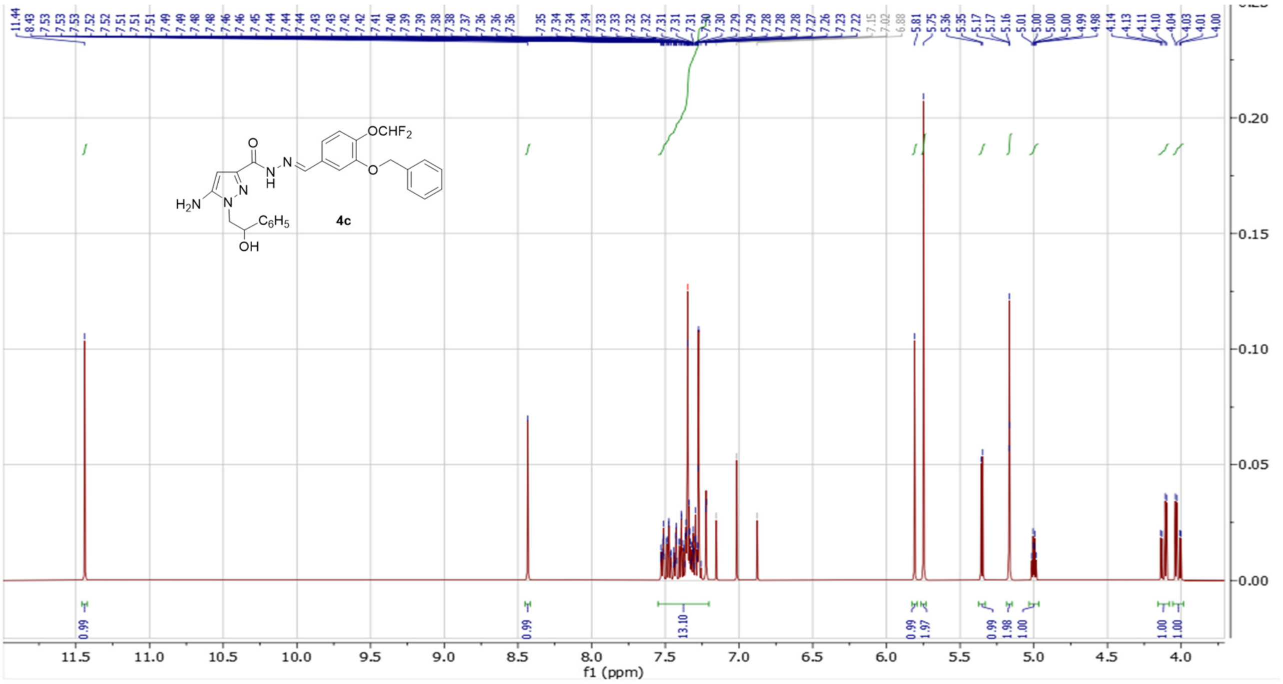

Figure S42: ^{13}C NMR (101 MHz) of compound **4c**

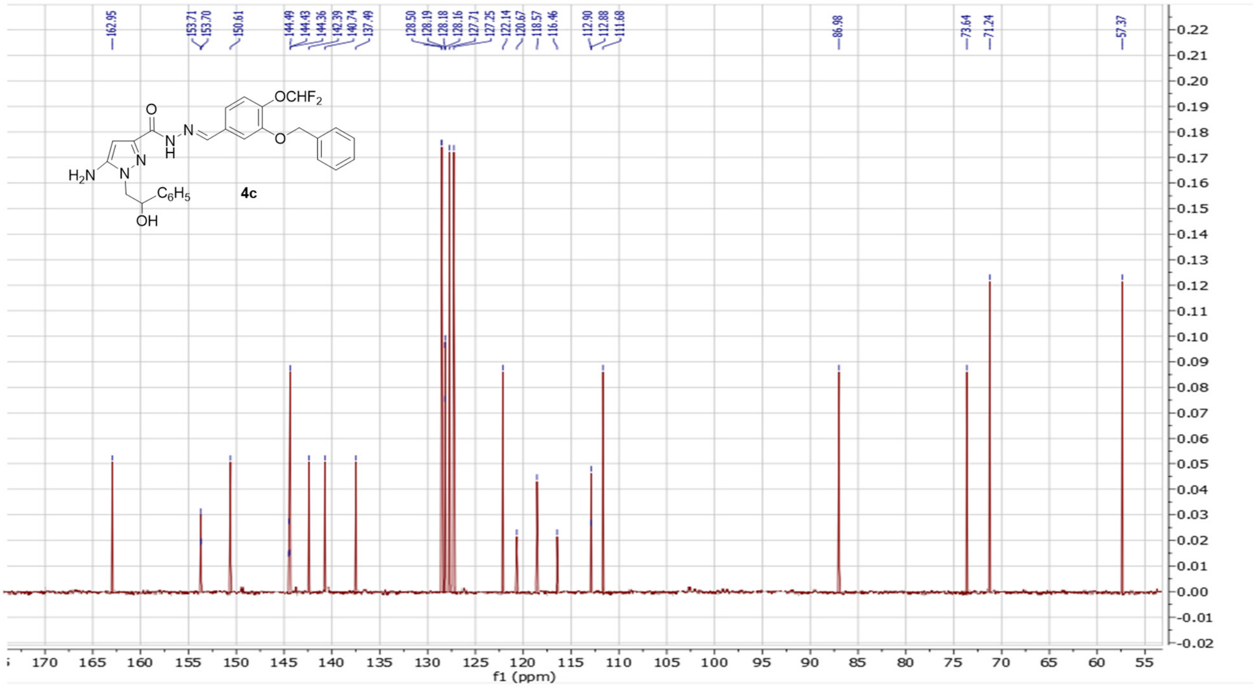

Supplement: Supplementary file 1 [file molecules-29-02298-s001.zip › molecules-2974194-supplementary.pdf]
